# Distinct discrepancy in breast cancer organoids recapitulation among molecular subtypes revealed by single‐cell transcriptomes analysis

**DOI:** 10.1002/ctm2.70023

**Published:** 2024-09-21

**Authors:** Ziqi Jia, Hengyi Xu, Yaru Zhang, Heng Cao, Chunyu Deng, Longchen Xu, Yuning Sun, Jiayi Li, Yansong Huang, Pengming Pu, Tongxuan Shang, Xiang Wang, Jianzhong Su, Jiaqi Liu

**Affiliations:** ^1^ Department of Breast Surgical Oncology, National Cancer Center/National Clinical Research Center for Cancer/Cancer Hospital Chinese Academy of Medical Sciences and Peking Union Medical College Beijing China; ^2^ State Key Laboratory of Molecular Oncology, National Cancer Center/National Clinical Research Center for Cancer/Cancer Hospital Chinese Academy of Medical Sciences and Peking Union Medical College Beijing China; ^3^ Eight‐year MD Program Peking Union Medical College Chinese Academy of Medical Sciences Beijing China; ^4^ Oujiang Laboratory (Zhejiang Lab for Regenerative Medicine, Vision, and Brain Health), Eye Hospital, Wenzhou Medical University Wenzhou China; ^5^ Tsinghua‐Peking Joint Center for Life Sciences Tsinghua University Beijing China; ^6^ Department of Head and Neck Surgical Oncology, National Cancer Center/National Clinical Research Center for Cancer/Cancer Hospital Chinese Academy of Medical Sciences and Peking Union Medical College Beijing China

Dear Editor,

Breast cancer organoids (BCOs) are increasingly recognised as crucial tools in personalised medicine,^1^ yet a significant gap remains between the need for precise drug sensitivity assessments and the biological disparities observed between BCOs and primary breast cancer (PBC) tissues.^2^, ^3^ Our extensive analysis of paired single‐cell RNA sequencing data has revealed a substantial preservation of molecular characteristics in hormone receptor‐positive (HR‐positive) and HER2‐positive breast cancers. However, in triple‐negative breast cancer (TNBC), we observed marked variability in cell subpopulations, likely influenced by oxygen‐enriched culture conditions.

To investigate the preservation of characteristics across different molecular subtypes of breast cancer, we cultured six BCOs representing three subtypes: two HR‐positive, two HER2‐positive, and two TNBCs derived from surgical samples without prior adjuvant treatments (for study design, see Figure S1; for images of successfully established organoids, see Figure S2; patient clinical characteristics are detailed in the Supplementary Table). Following establishment, single‐cell RNA sequencing was performed on matched PBCs and BCOs, yielding 66,920 quality‐controlled cells (Figure 1A; for contributions of samples, molecular subtypes, and sample sources, see Figure S3). Our analysis of cell type composition revealed a significant reduction in immune and stromal cells in BCOs compared to PBCs (adjusted *p* < 0.001; Figure 1B), while epithelial cells proportions nearly doubled (*p* = 0.031, median fold change = 0.96, IQR = 0.94‐1.78, Figure 1C). This suggests that organoid culture better preserves epithelial cells, and co‐culture systems are required for the preservation of the tumour microenvironment (TME).^4^ Further analyses demonstrated reductions in both the proportions and functionality of all immune and stromal cell subpopulations (Figure 1D‐F). Notably, both malignant and non‐malignant epithelial cells were amplified in BCOs while maintaining key functional characteristics (Figure 1G‐I; see Methods section in Supplementary Materials for malignancy determination). Thus, despite the observed differences in cell type distribution, these findings did not diminish the value of organoids as robust in vitro models for studying epithelial components of tumours.

**FIGURE 1:**
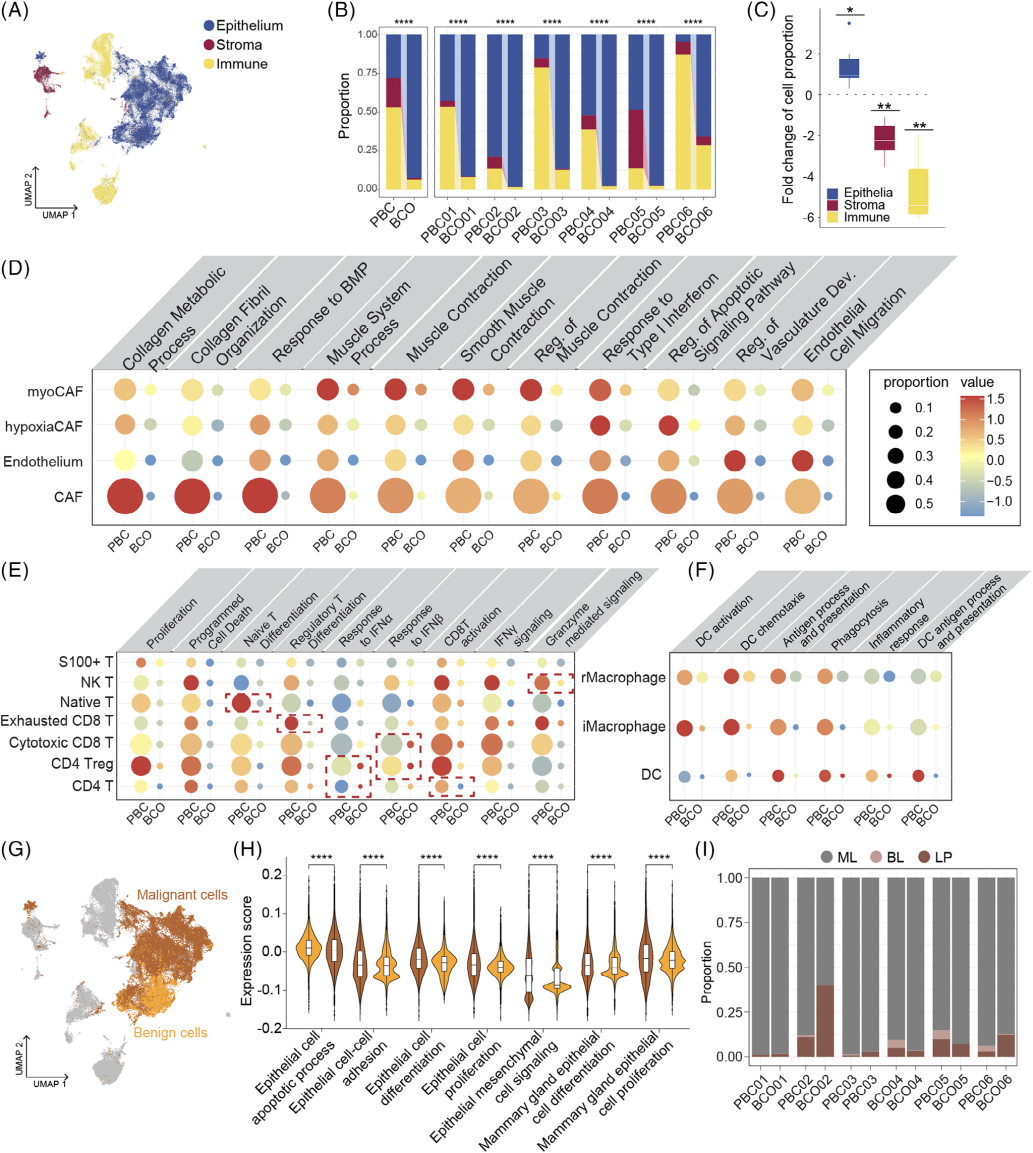
Global patterns of single‐cell transcriptome and breast cancer organoid preservation of primary breast cancer cell types. (A) Distribution of cell categories across cells from primary tumours and organoids. (B) Cell type ratios for the six primary tumour samples and matched six organoids. (C) Fold changes of different cell types between primary tumour samples and matched organoids. Asterisks indicate *p* Values determined by single‐sample *t*‐tests, where the mean fold change in cell type proportions across all samples was compared to zero (i.e., a fold change of zero). (D–F) Scores and proportions of different functions on each stromal cell (D), T cells (E), macrophages (F) subgroups between primary samples and organoids. (G) UMAP dimensionality reduction of cells and colours represent cell malignancy. (H) Violin plot showing the comparison of epithelium cell functions between malignant cells and benign cells. (I) Cell type ratios for primary tumour samples and organoids. Colours represent cell normal mammary. HR, hormone receptor; pos, positive; TNBC, triple‐negative breast cancer; PBC, primary breast cancer; BCO, breast cancer organoid; LP, luminal progenitor epithelium; BL, basal luminal epithelium; ML, mature luminal epithelium; UMAP, uniform manifold approximation and projection.

To assess genomic concordance in PDOs,^5^ we analysed copy number variation (CNV) as a genomic marker between BCOs and PBCs using both paired and unpaired comparisons. Our findings revealed that BCOs effectively preserved cellular‐level CNVs from PBCs in five out of six cases (Figure 2A), with an average retention rate of 71.6%. This preservation was particularly robust in HR‐positive breast cancer at 88.2%, though it was less pronounced in TNBC at 62.4% (Figure 2B and C). Moreover, BCOs demonstrated the ability to amplify both the magnitude and proportion of CNVs, including key oncogenes such as MYC on chromosome 8q and other tumour‐driver genes, resulting in increased levels and a higher proportion of cells with amplified or deleted CNVs (Figure S4). The lower CNV preservation observed in patient P06, associated with the TNBC subtype, highlighted the necessity for enhanced quality control in such cases. While previous studies have documented distinct DNA copy number retention in BCOs, our data further confirmed that organoids exhibit stronger and cleaner CNV signals, though retention patterns vary across different molecular subtypes.

**FIGURE 2:**
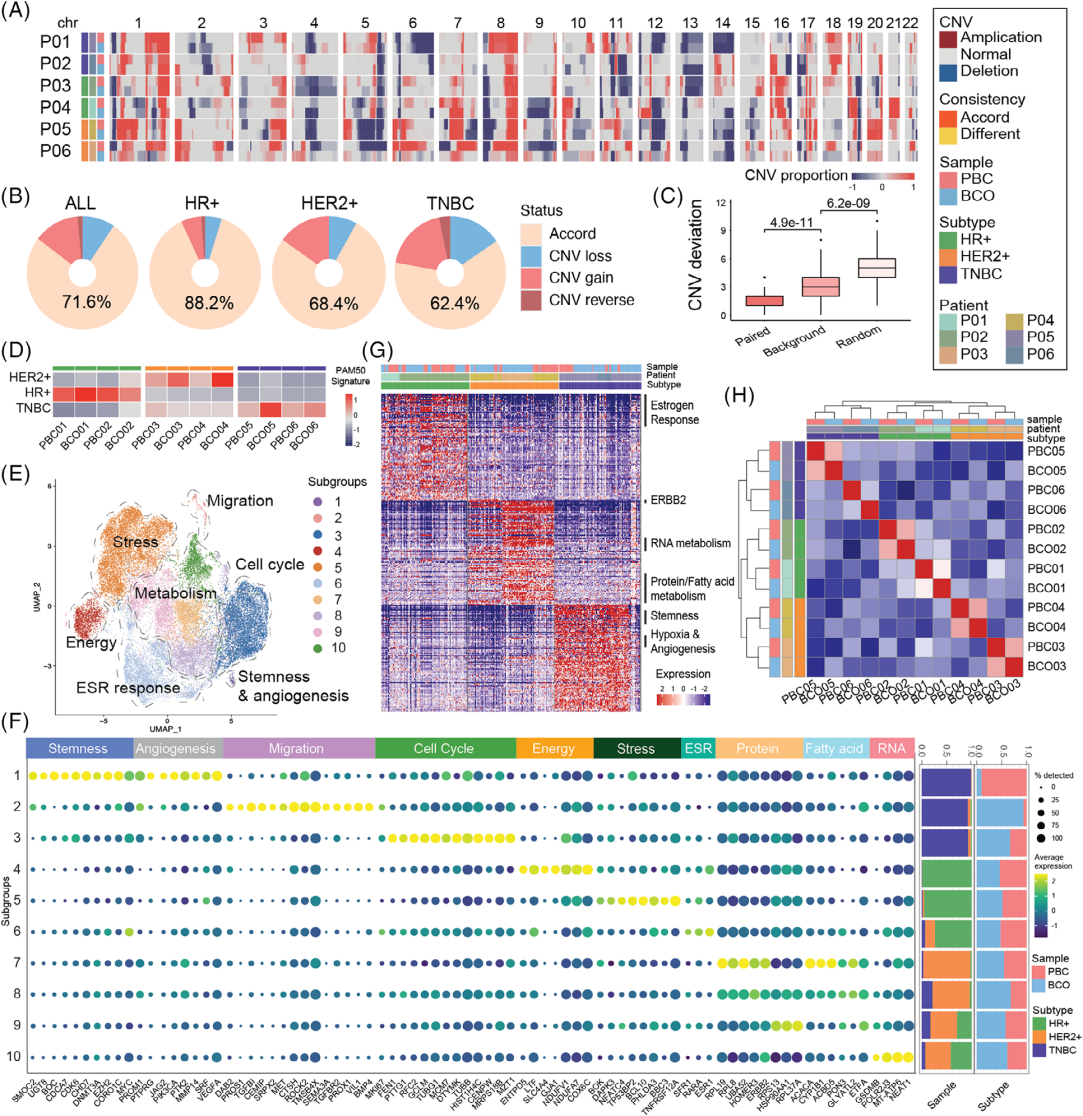
Molecular features preservation and discrepancy. (A) Heatmap showing CNVs on 22 chromosomes for each sample. (B) Proportions of different CNV changes on different tumour subtypes. *p* Values were determined by Student's *t*‐tests between CNV deviation scores from different compare groups. (C) CNV deviation scores of paired samples, background samples, and random controls. Details in Methods. (D) Heatmap illustrating PAM50 gene scores on 3 subtypes in each sample. (E) UMAP representation of functional subgroups across malignant cells. (F) Expression of selected genes on each functional cell subgroup. (G) Column plots exhibiting cell ratios from different subtypes or sources in each cell subgroup are on the right. Heatmap demonstrating expression of selected genes in malignant cells of different molecular subtypes. (H) Heatmap and unsupervised clustering for the correlation patterns based on subtype marker genes between samples. P, patient; BC, breast cancer; HR, hormone receptor; pos, positive; TNBC, triple‐negative breast cancer; PBC, primary breast cancer; BCO, breast cancer organoid; CNV, copy number variation; UMAP, uniform manifold approximation and projection.

Gene expression profiling was conducted to assess whether BCOs retain key biological characteristics of PBCs. The PAM50 assay confirmed that BCOs accurately preserved the molecular subtypes of the original samples (Figure 2D).^6^ However, in HR‐positive breast cancer, both ESR1 expression and the proportion of cells with high ESR1 expression were significantly reduced in BCOs compared to PBCs (*p* < 0.001, Figure S5). For HER2‐positive breast cancer, 99.5% of cells in both PBCs and BCOs exhibited elevated ERBB2/HER2 expression, although the expression levels were higher in PBCs (*p* < 0.05, Figure S5). In the case of TNBC, claudin‐low cells were similarly proportioned in both sources, but BCOs demonstrated increased MKI67 expression levels, indicating higher proliferation activity (Figure S5).

To evaluate cellular heterogeneity and the preservation of key cell clusters in BCOs across different molecular subtypes, we employed Seurat to cluster cells into 11 functional subgroups (Figure 2E).^7^ Analysis of molecular subtyping and the origin of these subgroups revealed that the estrogen receptor response subgroup, which predominated in HR‐positive breast cancer, and the metabolism subgroups, which were prominent in HER2‐positive breast cancers, were highly preserved in the organoids. In TNBC, the migration subgroup was both preserved and significantly expanded in BCOs (subgroup 2, Figure 2F); however, the subgroup characterised by high stemness, angiogenesis, and hypoxia expression (subgroup 1, Figure 2F) was almost entirely lost in BCOs. A differential expression and functional scoring heatmap demonstrated that, under unsupervised clustering, cells from BCOs and PBCs of HR‐positive and HER2‐positive breast cancers intermingled well within their respective molecular subtypes, confirming that organoids effectively retain molecular subtype characteristics in these two subtypes (Figure 2G and H). In contrast, the key features of TNBC in PBCs were poorly recapitulated in their matched BCOs.

To further investigate the loss of stemness and hypoxia‐related characteristics between PBCs and BCOs, we identified the stemness subgroup within TNBC cells and validated its stemness using established cancer stem cell markers CD44 and ALDH1A2 through functional scoring (Figure 3A).^8^ TNBC organoid stem cells exhibited decreased expression in stemness‐related pathways and increased MKI67 expression compared to PBCs (Figure S6A and B). Pseudotime evolutionary analysis revealed a cell transition trajectory where cells from BCOs predominantly congregate near the terminal stages (Figure 3B). Similarly, CNV‐based evolution analysis indicated that as the tumour evolved, a greater proportion of cells originated from BCOs (Figure 3C). Given that BCOs were cultured from surgical samples collected from PBCs, the loss of stemness cell subgroup in TNBC may be attributed to the prolonged culture conditions.

**FIGURE 3:**
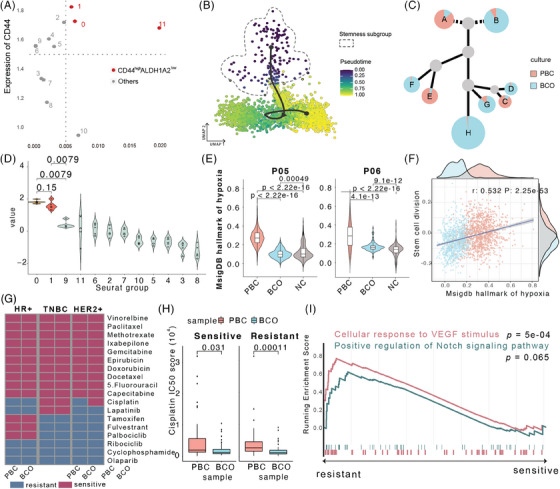
Variance between breast cancer organoids and primary triple‐negative breast cancer in stemness, hypoxia‐related pathways, and drug sensitivity. (A) Dot plot exhibiting the expression of CD44 and ALDH1A2 in each cell cluster. Red dots representing CD44‐high, ALDH1A2‐low cell clusters. (B) UMAP representation of pseudotime analysis across TNBC malignant cells. (C) Tumour phylogenetic trees of patient 03. Colours indicate cells from PBC (red) or BCO (blue), while dot size represents cell numbers. (D) Violin plot illustrating the levels of different hypoxia scores between TNBC malignant cell subgroups. *p* Values were determined by Student's *t*‐tests between hypoxia scores from Seurat clusters. (E) Violin plot showing the hypoxia scores between non‐hypoxia groups, PBC hypoxia groups, and BCO hypoxia groups. *p* Values were determined by Student's *t*‐tests between hypoxia scores from different cell groups. (F) Scatter plot and linear regression hypoxia hallmark score and stem cell division score in TNBC cancer stem cells; Density plot demonstrating the distribution of cells from BCO (blue) or PBC (red) are in upper and right. *r* stands for Pearson correlation ecoefficiency, while the *p* value was calculated by linear regression *t*‐test. (G) Heatmap using the drug sensitivity spectrums between primary tumour samples and organoids. (H) Boxplots exhibiting inferred IC50 between cells from PBC and BCO in the cisplatin‐sensitive cell group (left) or cisplatin‐resistant cells group (right), while a higher IC50 indicates a stronger drug resistance. *p* Values were determined by Student's *t*‐tests between inferred IC50 from PBC and BCO. (I) GSEA enrichment plot for the differential expression genes between drug‐resistant and drug‐sensitive groups in TNBC malignant cells. P, patient; BC, breast cancer; HR, hormone receptor; pos, positive; TNBC, triple‐negative breast cancer; PBC, primary breast cancer; BCO, breast cancer organoid; CNV, copy number variation; UMAP, uniform manifold approximation and projection.

To explore the influential role of hypoxia, we analysed hypoxia‐related pathway expression using a previously established hypoxia score, which was found to be lower in BCO‐derived cells (Figure 3E). Further correlation analysis revealed a significant positive relationship between hypoxia markers and stem cell division scores (*r* = 0.532, *p *< 0.001; Figure 3F). Notably, cancer cells derived from BCOs exhibited a reduced presence of both stemness and hypoxia features (Figure 3F), such finding was also observed in T cells (Figure S7). This finding could be attributed to the discrepancy in oxygen levels, with breast cancer tissues having a partial pressure of oxygen (PO_2_) of approximately 10 mmHg, compared to the 150 mmHg typically found in organoid cultures.^9^ These results highlighted the critical role of hypoxia in maintaining cancer cell stemness, offering a plausible explanation for the reduced stemness observed in BCOs.

Drug sensitivity consistency is fundamental to the clinical application of BCOs. To assess this, we performed drug sensitivity analysis using OncoPredict,^10^ which demonstrated that BCOs generally retained the drug sensitivity profiles of their corresponding primary tumours across breast cancer subtypes, with the exception of TNBC, where BCOs exhibited increased sensitivity to cisplatin compared to PBCs (Figure 3G). To further investigate the causes of this discrepancy in cisplatin response, malignant TNBC cells were categorised into drug‐sensitive and drug‐resistant groups based on the cluster‐based OncoPredict outcomes. A comparison of half‐maximal inhibitory concentration (IC50) between these groups revealed that PBC‐derived cells exhibited higher resistance levels in both sensitivity (*p* = 0.031) and resistant (*p* = 0.001) groups (Figure 3H, external validation see Figure S8). Drug‐resistant cells, in particular, showed elevated expression of the stemness‐associated Notch pathway and the hypoxia‐associated VEGF pathway, with significant correlations suggesting a link between hypoxia, stemness, and cisplatin resistance (Figure 3I).

Several limitations must be acknowledged. First, the inference of single‐cell level CNVs was based on gene expression data, which may compromise their accuracy. Additionally, drug sensitivity was accessed solely through in silico analysis, necessitating further functional experiments in vitro to validate the observed discrepancies in drug response between PBCs and BCOs.

In conclusion, we identified significant preservation of molecular characteristics in BCOs, alongside critical discrepancies, notably the loss of specific cellular subgroups associated with stemness and hypoxia, factors crucial for accurate drug response predictions. Our findings suggest that the current organoid culture conditions markedly influence cellular composition, thereby impacting the clinical applicability of PDOs in treatment strategies. While the existing culturing methods effectively preserve characteristics in HR‐positive and HER2‐positive subtypes, they result in the loss of stemness in TNBC, which may compromise the utility of BCOs for monitoring drug sensitivity in this subtype.

## AUTHOR CONTRIBUTIONS

Ziqi Jia collected the samples, performed the analyses, and wrote the manuscript. Hengyi Xu performed the analyses and prepared the figures. Yaru Zhang conducted the external validation. Heng Cao, Chunyu Deng, Longchen Xu, Yuning Sun, Jiayi Li, Yansong Huang, Pengming Pu, and Tongxuan Shang participated in sample collection and process and data preprocessing. Jiaqi Liu conceived the project and designed the research. Jiaqi Liu, Jianzhong Su, and Xiang Wang were responsible for the study supervision and manuscript revision. All authors approved the final version of the manuscript.

## CONFLICT OF INTEREST STATEMENT

The authors declare no potential conflicts of interest

## FUNDING

This work was supported by National Natural Science Foundation of China (Grant No. 82272938 to J. Liu), Beijing Nova Program (Grant No. 20220484059 to J. Liu), CAMS Innovation Fund for Medical Sciences (Grant No. 2021‐I2M‐1‐014 to J. Liu), Beijing Hope Run Special Fund (Grant No. LC2020B05 to J. Liu), and Beijing Science and Technology Innovation Foundation for University or College Students (Grant No. 2022zglc06074 to HX).

## ETHICS STATEMENT

This study has been approved by the Institutional Review Board (IRB) of Cancer Hospital, Chinese Academy of Medical Sciences (NCC20230‐241).

## Supporting information



Supporting Information

Supporting Information

## Data Availability

All data are available in the main text or the Supplementary Materials. The supplementary data supporting this study's findings are available in the Supplementary Materials. Deidentified participant data and analytic code are available upon reasonable request to Dr. Jiaqi Liu (j.liu@cicams.ac.cn).
