# Supplementary material for: Distinct discrepancy in breast cancer organoids recapitulation among molecular subtypes revealed by single‐cell transcriptomes analysis
Source: Clin Transl Med. 2024 Sep 21;14(9):e70023. doi: 10.1002/ctm2.70023 (PMC11416080; doi:10.1002/ctm2.70023)
Supplement: Supplementary file 2 — Supporting Information [file CTM2-14-e70023-s001.docx]

**Supplementary Online Content**

**Jia *et al*. Evaluating the Accuracy of Breast Cancer Organoids in Mirroring Primary Tumor Heterogeneity at Single-cell Resolution**

[Supplementary Methods: 3](#_Toc175432188)

[*Experimental Design* 3](#_Toc175432189)

[*Participants and Clinical Evaluation* 3](#_Toc175432190)

[*Patient-derived breast cancer organoid culture* 3](#_Toc175432191)

[*Single-cell RNA-sequencing library construction* 3](#_Toc175432192)

[*FASTQ quality control and reads mapping* 4](#_Toc175432193)

[*Expression matrix pre-processing* 4](#_Toc175432194)

[*Nonlinear dimensional reduction on the transcriptome expression matrix* 4](#_Toc175432195)

[*Cell type determination and functional enrichment* 4](#_Toc175432196)

[*Distinguishing malignant from nonmalignant epithelial cells based on CNVs* 5](#_Toc175432197)

[*Differential expression and functional score calculation* 5](#_Toc175432198)

[*Breast cancer subtype assignment* 6](#_Toc175432199)

[*Copy number variation inference* 6](#_Toc175432200)

[*CNV deviation score calculation* 6](#_Toc175432201)

[*Tumor phylogenetic reconstruction* 6](#_Toc175432202)

[*Functional subgroup analysis* 6](#_Toc175432203)

[*Trajectory analysis* 7](#_Toc175432204)

[*Prediction of drug sensitivity based on scRNA-Seq* 7](#_Toc175432205)

[*Statistical analysis* 7](#_Toc175432206)

[Supplementary Table. Clinical characteristics of the involved breast cancer patients 8](#_Toc175432207)

[Figure S1. Graphic Abstract. 9](#_Toc175432208)

[Figure S2. Zoomed-in bright-field images of patient-derived tumor organoids. 10](#_Toc175432209)

[Figure S3. Global pattern of cell type annotations. 11](#_Toc175432210)

[Figure S4. Heatmap using CNVs on key tumor-driver oncogenes for each sample. 12](#_Toc175432211)

[Figure S5. Key biological characteristics for each molecular subtype can be maintained in tumor organoids. 13](#_Toc175432212)

[Figure S6. Further function analysis on cancer stem cells in TNBC. 14](#_Toc175432213)

[Figure S8. Function analysis on hypoxia for tumor microenvironment in triple-negative breast cancer. 15](#_Toc175432214)

[Figure S8. Drug resistance validation with external database 16](#_Toc175432215)

[Figure S9. Single-cell data quality control and batch elimination. (Cited in Supplementary Methods) 17](#_Toc175432216)

[Figure S10. Cell type annotation. (Cited in Supplementary Methods) 18](#_Toc175432217)

[Figure S11. Stromal cells in primary breast cancer and breast cancer organoids. (Cited in Supplementary Methods) 19](#_Toc175432218)

[Figure S12. Patient-derived organoids preserved immune cells of different subtypes. 21](#_Toc175432219)

[Figure S13. Patient-derived organoids preserved immune cells with reduced quantity and functions. 22](#_Toc175432220)

[Figure S14. Benign-malignant distinction of epithelium cells. (Cited in Supplementary Methods) 25](#_Toc175432221)

[Figure S15. Analysis on benign epithelium cells. (Cited in Supplementary Methods) 26](#_Toc175432222)

[Figure S16. Hormone receptor-positive breast cancer function analysis revealed preserved cell stemness and cell trajectory in tumor organoids. 27](#_Toc175432223)

[Figure S17. HER2-positive breast cancer function analysis revealed preserved cell stemness and cell trajectory in tumor organoids. 29](#_Toc175432224)

[Figure S18. Global CNV patterns in malignant epithelium cells 31](#_Toc175432225)

This supplementary material has been provided by the authors to give readers additional information about their work.

# Supplementary Methods:

## *Experimental Design*

To investigate the recapitulation of primary breast cancer in patient-derived organoids, a systematic evaluation was conducted involving six patients representing three molecular subtypes. The patients were enrolled for tumor resection and subsequent PDO construction following pathology-based diagnosis. After seven days of culture, scRNA-seq was performed on both PBCs and matched PDOs. The analysis of single-cell transcriptome profile focused on three main aspects: the tumor microenvironment (including the cell composition and function of immune/stromal cells), the evolution of malignant cells (CNV deduction and trajectory analysis), and the preservation of malignant cell function (including molecular subtype features, cell stemness, and hypoxia). Overall study design is shown in Fig S1.

## *Participants and Clinical Evaluation*

Six patients with BC were enrolled at the Cancer Hospital of the Chinese Academy of Medical Sciences (CHCAMS). The diagnosis of each patient was based on the pathology results. The clinical staging was determined according to the 8th edition of the classification for breast cancer by the American Joint Commission of Cancer (AJCC)^7^. Meanwhile, the molecular subtype was divided according to the pathologic criteria for HR (including ER and PR) and HER2^6^. The ER, PR, HER2, and Ki-67 were measured by immunohistochemistry. In total, we enrolled two patients with HR-positive/HER2-negative BC (P01 and P02), two with HR-negative/HER2-positive BC (P03 and P04), and two with TNBC (P05 and P06). Patients’ clinical information was collected (Table S1). All participants provided informed consent for the study. Isolated from resected tumors, tumor tissues were stored in MACS^®^ Tissue Storage Solution (Miltenyi Biotec Inc., USA) at 4 °C.

## *Patient-derived breast cancer organoid culture*

Isolated tumor epithelium cells were cultured as organoids, as described by Sato *et al.*^17^. After washing in cold wash buffer thrice for 5 minutes, breast cancer tissues were minced into fragments smaller than 1 mm3, followed by digestion with digestion buffer containing 500 U/mL collagenase IV, 1.5 mg/mL collagenase II, 20 mg/ml hyaluronidase, 0.1 mg/mL dispase type II, 10 mM ROCK Inhibitor Y-27,632 and 1% fetal bovine serum at 37 °C for 30 min. Filters of 100 and 70 um were used to filter the tissue-cell mixture. About 1.5 × 10^6^ single cells/mL were washed and then embedded in 20 µL Matrigel in a 48-well plate. Using OneCULTarTM intestinal tumor culture medium, they were refreshed every 3-4 days and passaged every 7 days.

## *Single-cell RNA-sequencing library construction*

BC specimens were minced and washed in ice-cold RPMI1640. The stromal gel was stripped for organoids, and cells were washed in ice-cold RPMI1640. Tissue was then digested with Sigma Collagenase II and DNase I and lysed with red blood cell lysis solution. Live-dead dye was used for deal cell exclusion. Washed twice, digested cells were resuspended in 1 x PBS with 0.04% bovine serum albumin, and a 10x Genomics Chromium Controller was used for single-cell partitioning and Gel Bead-in-Emulsion (GEM) generation. cDNA libraries were checked for quality using an Agilent 2100 Bioanalyzer. For each library, adapters were ligated, barcodes were created, and fragment sizes were assessed. Samples were then pooled and sequenced using a Novaseq 6000 S4 flow cell using 10× Genomics Chromium Controller protocol (version CG000204).

## *FASTQ quality control and reads mapping*

The Cell Ranger pipeline (version 5.0.1) was used for each scRNA-seq data set to perform the alignment against the human reference genome hg38, filter the alignment and count barcodes and Unique molecular identifiers (UMIs)^48-50^.

## *Expression matrix pre-processing*

Standard pre-processing and downstream analyses were utilizing the R package *Seurat* (version 4.1.1)^51^. The parameters of filtering for cells were as follows: a low.threshold of 200 and a high.threshold of 7000 for gene numbers in each cell; a low.threshold of 500 for the number of counts per cell; a high.threshold of 20% for mitochondrial genes; a high.threshold of 20% for ribosomal genes percentage; a min.cell of 3. Matrices were normalized by the LogNormalize method using a scale factor of 10,000. The cell cycle scores of individual cells were calculated based on the expression of G2/M and S phase markers by the *AddModuleScore* function^51^. Normalized matrices were scaled by the *SCTransform* function by regressing out the total cellular UMI counts, mitochondrial gene percentage, and cell cycle scores.

## *Nonlinear dimensional reduction on the transcriptome expression matrix*

Highly variable genes were calculated using the *FindVariableGenes* function and sent to *RunPCA* for PCA linear dimensionality reduction, and the first 50 PCs were selected^52^. Function *RunHarmony* was used for batch elimination between different samples. After batch elimination, unified manifold approximation and projection (UMAP) was performed using the function *RunUMAP*. Cell clustering was completed using *FindNeighbors* and *FindClusters,* with the resolution parameter set to 0.5. All 12 scRNA-seq profiles were merged and normalized together, and no batch effects were detected by PCA analysis after the Harmony algorithm (***Fig. S9***).

## *Cell type determination and functional enrichment*

Differentially expressed genes (DEGs) were identified by the *FindAllMarkers* function using the Wilcoxon test. Gene Ontology (GO) analyses were conducted using the R package *clusterProfiler* (version 4.0.5)^53^ based on the top 300 genes for each cell cluster. Furthermore, all cell clusters were automatically annotated using the *SingleR* (version 1.6.1)^54^. Cell types were ultimately annotated based on established marker genes, functional enrichment results, and the putative cell type information from *SingleR*. To further compare cell component differences between BCOs and PBCs, we first conducted unsupervised clustering of all cells from the merged profile. Three major cell clusters were identified as epithelial cells (*EPCAM^+^*, *KRT7^+^*, *KRT8^+^*, *KRT19^+^*, *CD24^+^*), immune cells (*PTPRC^+^*, *CD3D^+^*, *CD3E^+^*, *CD4^+^*, *NKG7^+^*, *CD8A^+^*, *CD14^+^*, *CD69^+^*), and stroma cells (*PECAM1^+^*, *PDPN^+^*, *FAP^+^*, *ACTA2^+^*, *COL1A1^+^*, *TAGLN^+^*) by established marker genes (***Fig. S10***).^9^

Stroma cells were further cluster into myofibroblastic cancer associated fibroblasts (myoCAFs: *MCAM*^+^, *ACTA2*^+^, *TAGLN*^+^, *MYLK*^+^, *CAV1*^+^), hypoxia-related cancer associated fibroblasts (hypoxiaCAFs: *VEGFA*^+^, *ERO1A*^+^, *PGK1*^+^, *ENO1*^+^, *BNIP3L*^+^, *FAM162A*^+^, *BNIP3*^+^), cycling cancer associated fibroblasts (cycling CAFs: *FAP*^+^, *COL1A1*^+^, *PDGFRA*^+^, *PDPN*^+^, *MKI67*^+^, *AURKA*^+^, *CDKN3*^+^, *CDK1*^+^), other cancer associated fibroblasts (CAFs: *FAP*^+^, *COL1A1*^+^, *PDGFRA*^+^, *PDPN*^+^), and endothelia (*VWF*^+^, *ICAM2*^+^, *PLVAP*^+^, *PECAM1*^+^) by established marker genes (***Fig. S11***).

Immune cells were also further clustered into NK cells (*NKG7*^+^), CD8+ T cells (*CD3D*^+^, *CD3E*^+^, *CD8A*^+^), CD4+ T cells (*CD3D*^+^, *CD3E*^+^, *CD4*^+^), cell cycle T cells (*MKI67*^+^), B cells (*CD79A*^+^, *CD19*^+^, *MS4A1*^+^), plasma cells (*CD79A*^+^), monocytes/macrophages (*MRC1*^+^, *FCGR3A*^+^, *CD68*^+^, *CD163*^+^), dendritic cells (*MRC1*^+^, *FCGR3A*^+^, *CD68*^+^, *CD163*^+^, *CLEC10A*^+^, *CD1C*^+^, *HLA-DQA2*^+^, *HLA-DRB5*^+^), mast cells (*MS4A2*^+^, *GATA2*^+^, *KIT*^+^, *CPA3*^+^, *IL1RL1*^+^) by established marker genes (***Fig. S12***).

Macrophages were further clustered into dendritic cells, inflammatory macrophages, and resting macrophages (see markers in ***Fig. S13D***). T cells were further clustered into naïve T cell, cytotoxic CD8+ T cells, CD4+ T cells, exhausted CD8+ T cells, CD4+ regulatory T cells, and S100+ T cells (see markers in ***Fig. S13I***).

## *Distinguishing malignant from nonmalignant epithelial cells based on CNVs*

Estimates of CNV signals were achieved using the R package *inferCNV* (version 1.8.1)^22^. This package computed CNVs from gene expression levels. Following the official tutorial, macrophages were selected as reference cells (***Fig. S14C***). The CNV score for each cell was calculated by summing CNV levels from all chromosome regions. CNV levels (from 0 to 2) were the absolute values of inferred CNV status (from -2 to 2). *InferCNV* also outputs a hierarchical dendrogram representing the clustering results that divide epithelium cells into 31 clusters by default parameters. Epithelium cell clusters with a CNV score > 6000 were considered malignant cells (***Fig. S14A***). Validation is performed by tumor-driver gene expression (***Fig. S14D***), epithelial cell function annotation, and unsupervised cluster analysis (***Fig. 1G***). Malignant cell clusters exhibited elevated tumor-driver gene expression and epithelial cell functions. Non-malignant cells were clustered together in an unsupervised manner, indicating consistency in their transcriptomic profile (***Fig. 1G***). Non-malignant epithelial cells displayed lower levels of epithelial-mesenchymal transition (EMT) and proliferation features when compared to their malignant counterparts. Unsupervised clustering analysis successfully classified benign epithelium cells into three distinct subtypes, namely luminal progenitor, basal luminal, and mature luminal cells (***Fig. S15B-C***). All three subtypes of non-malignant cells could be preserved in BCO, with luminal progenitor cells exhibiting the highest preservation rate (***Fig. S15A***).

## *Differential expression and functional score calculation*

After going through the same processing process with the same parameters, cells were analyzed by UMAP and clustered. DEGs and GO analyses were performed as mentioned above. We calculated functional scores using the *AddModuleScore* function in *Seurat* based on the specific gene signatures^51^.

## *Breast cancer subtype assignment*

The breast cancer subtype assignment score for each sample was calculated by *molecular.subtyping* function in R package *genefu* (version 2.24.2) with pam50.robust algorithm and default parameters^24^. The *genefu* package includes several algorithms for molecular subtype classification on breast cancer, which uses candidate genes to construct a subtype clustering model. RNA-seq data were fitted on the model to calculate subtypes scores.

## *Copy number variation inference*

CNV signals were determined using *inferCNV* hidden Markov model (HMM). The "subcluster" mode was used to determine clonal CNV changes. For simplification, Genome Reference Consortium Human Build 38 (GRCh38) cytoband information was used to convert CNVs into horizontal variations of p or q arms. Chromosome arms with a CNV proportion higher than 40% were annotated as gain or loss. For visualization, *Uphyloplot2* (version 2.3) is used for the autonomic construction of intra-patient phylogenetic tree^55^. GRCh38 gene position information was used to calculate the copy number variation of several tumor driver genes.

## *CNV deviation score calculation*

To quantificationally compare the CNV retention of tumor driver genes between PBC and matched BCO, we calculated the gene CNV deviation score (CNV_dev) based on the formula below:

$$\text{CNV\_dev}\text{ }\text{=}\sum_{\text{pair of samples}}^{\text{all pairs}} \left( \text{|CNV\_status}_{\text{primary}}\text{-}\text{CNV\_status}_{\text{organoid}}\text{|} \right)$$

CNV_status: loss = -1, gain = 1

In the “Paired” panel, the deviation score was calculated between every pair of PBC and BCO; In the “Background” panel, the score was calculated between PBCs and unpaired BCOs from the same subtype. In the “Random” panel, the scores were calculated randomly between PBCs and BCOs with random distributions.

## *Tumor phylogenetic reconstruction*

The tumor phylogenetic tree was constructed manually according to the CNV region inferred by the HMM following the principles below:

1. Cancer stem cells should have the lowest CNVs.
2. Along with evolution, the CNVs will always grow higher.
3. CNVs observed in primitive tumor clusters should be observed in more advanced cell clusters.

## *Functional subgroup analysis*

As different breast cancer subtypes had different characteristics, functional comparison is executed on the level of subtypes. In hypoxia analysis, subgroups representing the hypoxia feature were selected based on five hypoxia scores from the MsigDB database (Table S2)^29^. Functional scores were calculated using the *AddModuleScore* function as described above. Scores were centered by averaging values of all tumor samples. Subgroups with the highest hypoxia score were specified as hypoxia subgroups. In stemness analysis, the identification of cancer stem cell groups also considered cancer stem cell markers and trajectory analysis results.

## *Trajectory analysis*

R package *dyno* (version 0.1.2) was used to infer cell differentiation trajectory^56^. Pseudotime analysis was performed based on the inferred trajectory to explore impressive translational cell relationships. This package comprehensively considered various requirements, including trajectory discontinuity, topology structure, and bifurcated trajectory, to select the most suitable trajectory-construction algorithm by function *guidelines_shiny*. Following the results, trajectory analysis on TNBC subtypes used the *dpt* method; trajectory analysis on HR-positive subtypes used *mst* method; trajectory analysis on HER2-positive subtypes used the *comp1* method. Trajectory inference was achieved by the *infer_trajectory* function. For gene-level pseudotime analysis, the *calculate_overall_feature_importance* function was performed.

## *Prediction of drug sensitivity based on scRNA-Seq*

Drug sensitivity inferences on malignant cells were achieved using the *calcPhenotype* function in the R package *oncopredict* (version 0.2) with a removeLowVaryingGenes of 0.8 and a minNumSamples of 100 ^32^. *Oncopredict* constructed a regression model based on databases of drug sensitivity data (IC50) and paired RNA-seq data to predict IC50 values. The estimation of drug sensitivity in single-cell data can be achieved through utilizing *oncopredict* following group averaging into bulk data. Cells with IC50 higher than 1000 were considered resistant cells.

## *Statistical analysis*

Numerical results were reported as means ± SEM as indicated. The Mann-Whitney test assessed Statistical significance between means (*GraphPad Prism 9.0*; *GraphPad* Software, www.graphpad.com). *P* < 0.05 was defined as the threshold for statistical significance. The Benjamini-Hochberg (BH) method was used for *P* value correction in multiple testing.

# Supplementary Table. Clinical characteristics of the involved breast cancer patients

| **Patient ID** | **Age** | **Gender** | **Stage** | **TNM** | **Subtype** | **ER** | **PR** | **HER2^✢^** | **Ki67%** |
| --- | --- | --- | --- | --- | --- | --- | --- | --- | --- |
| P01 | 69 | F | IIIA | T3N1aM0 | HR+/HER2- | 80% | 60% | - | 20% |
| P02 | 49 | F | IIA | T2N0M0 | HR+/HER2- | 80% | 60% | - | 20% |
| P03 | 76 | F | IIB | T2N1aM0 | HR-/HER2+ | 0 | 0 | + | 60% |
| P04 | 63 | F | IIA | T2N0M0 | HR-/HER2+ | 0 | 0 | + | 30% |
| P05 | 57 | F | IIIC | T2N3M0 | TNBC | 0 | 0 | - | 10% |
| P06 | 53 | F | IIA | T2N0M0 | TNBC | 0 | 0 | - | 60% |

Abbreviations: ID identity document; TNM tumor node metastasis; ER estrogen receptor; PR progesterone receptor; HER2: human epidermal growth factor receptor 2.

✢ The HER2 status mentioned is summarized based on both IHC and FISH results.


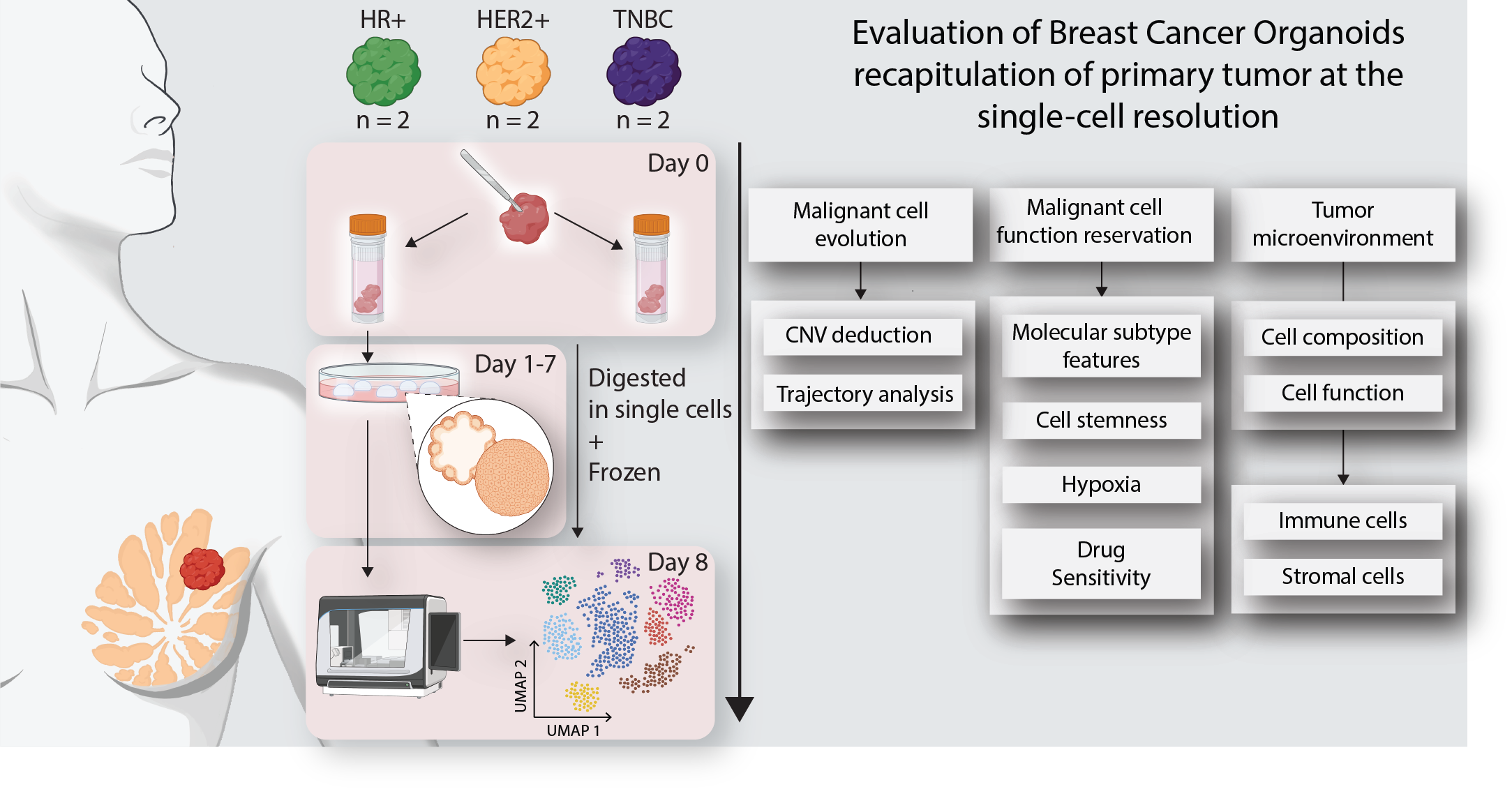


# Figure S1. Graphic Abstract.

Schematic workflow of patient enrollment, organoid culture, and single-cell RNA sequencing data generation.


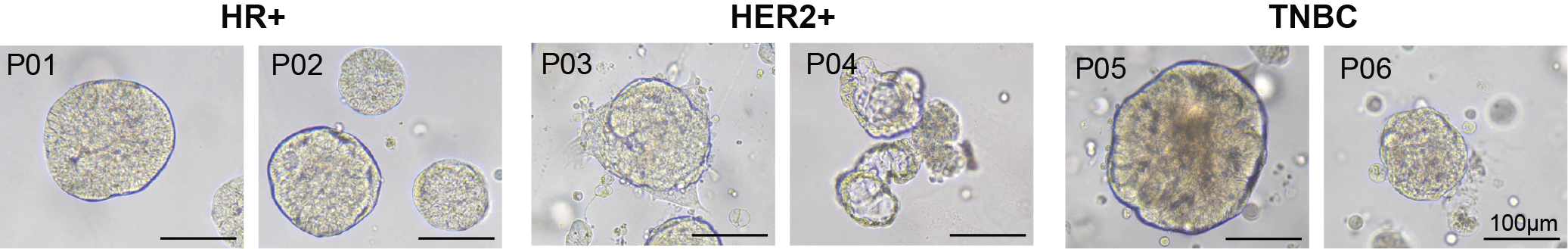


# Figure S2. Zoomed-in bright-field images of patient-derived tumor organoids.

Abbreviations: HR+, hormone receptor positive subtype; HER2+, HER2-positive subtype; TNBC, triple negative breast cancer subtype. P01-P06: patient id.


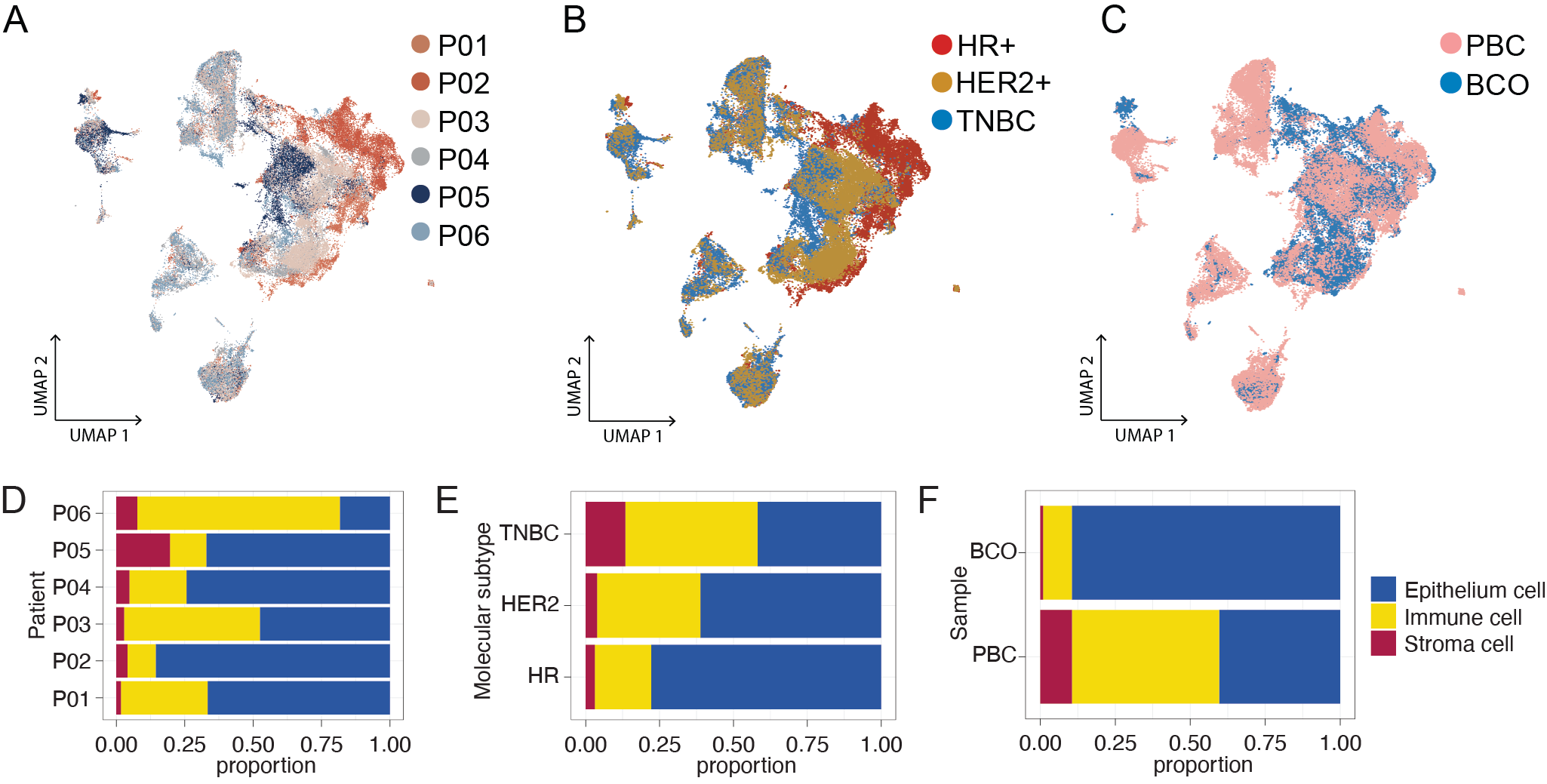


# Figure S3. Global pattern of cell type annotations.

**(A-C)** Distribution of patients (A), molecular subtypes (B), and sample sources (PBCs or BCOs) (C) across cells from primary tumors and organoids in UMAP. **(D-F)** Cell type ratios for samples (D), subtypes (E), and sources (F). Colors represent for cell types.

Abbreviations: PBC, primary breast cancer; BCO, breast cancer organoid; HR+, hormone receptor positive subtype; HER2+, HER2-positive subtype; TNBC, triple negative breast cancer subtype. P01-P06: patient id; UMAP, uniform manifold approximation and projection.


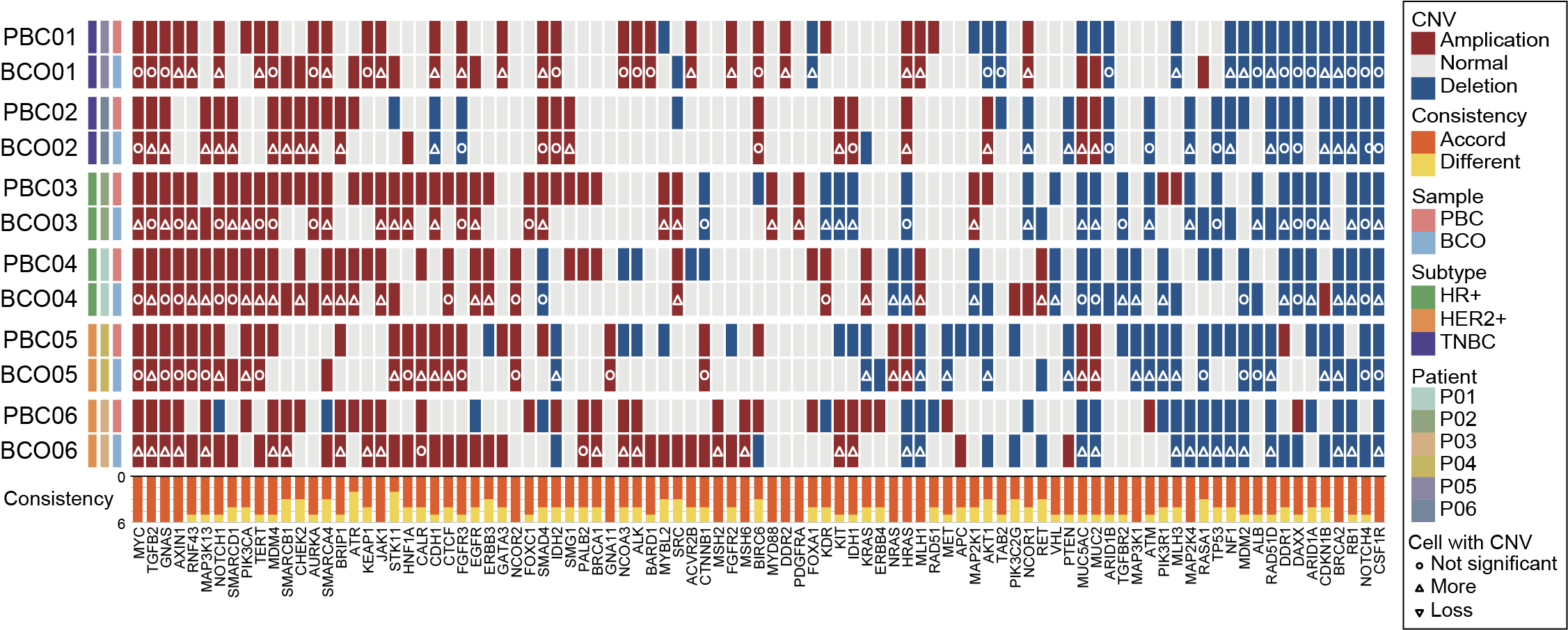


# Figure S4. Heatmap using CNVs on key tumor-driver oncogenes for each sample.

Each icon represents the change of a cell proportion with a specific gene CNV: circle stands for no significant change; triangle stands for significant proportion change (Chi-square test, *P* < 0.05).

Abbreviation: P, patient; BC, breast cancer; HR, hormone receptor; pos, positive; TNBC, triple-negative breast cancer; PBC, primary breast cancer; BCO, breast cancer organoid; CNV, copy number variation.
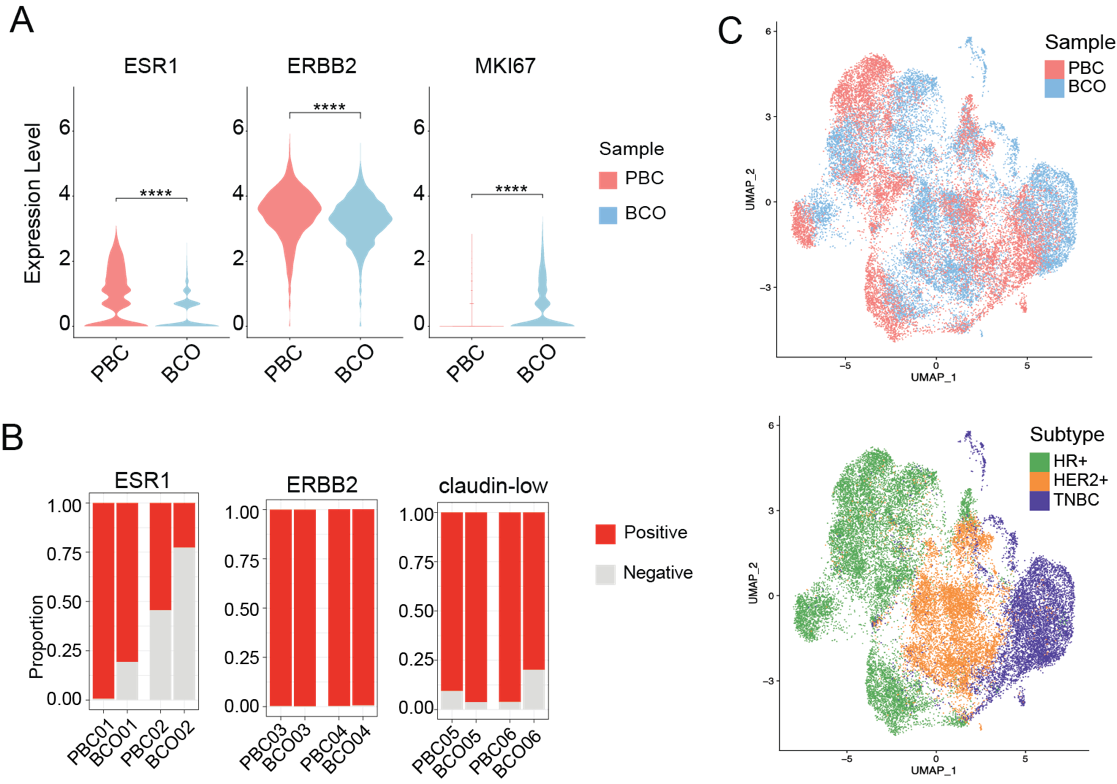


# Figure S5. Key biological characteristics for each molecular subtype can be maintained in tumor organoids.

(**A**) Expression comparisons of different subtype markers between primary tumor and matched organoids. *P* values were determined by Student’s t-tests between gene expressions from PBC and BCO. (**B**) Cell ratio comparisons of different key subgroups between primary tumor and matched organoids. (**C**) UMAP representation of subtypes and sources across malignant cells.

Abbreviations: P, patient; BC, breast cancer; HR, hormone receptor; pos, positive; TNBC, triple-negative breast cancer; PBC, primary breast cancer; BCO, breast cancer organoid; UMAP, uniform manifold approximation and projection.


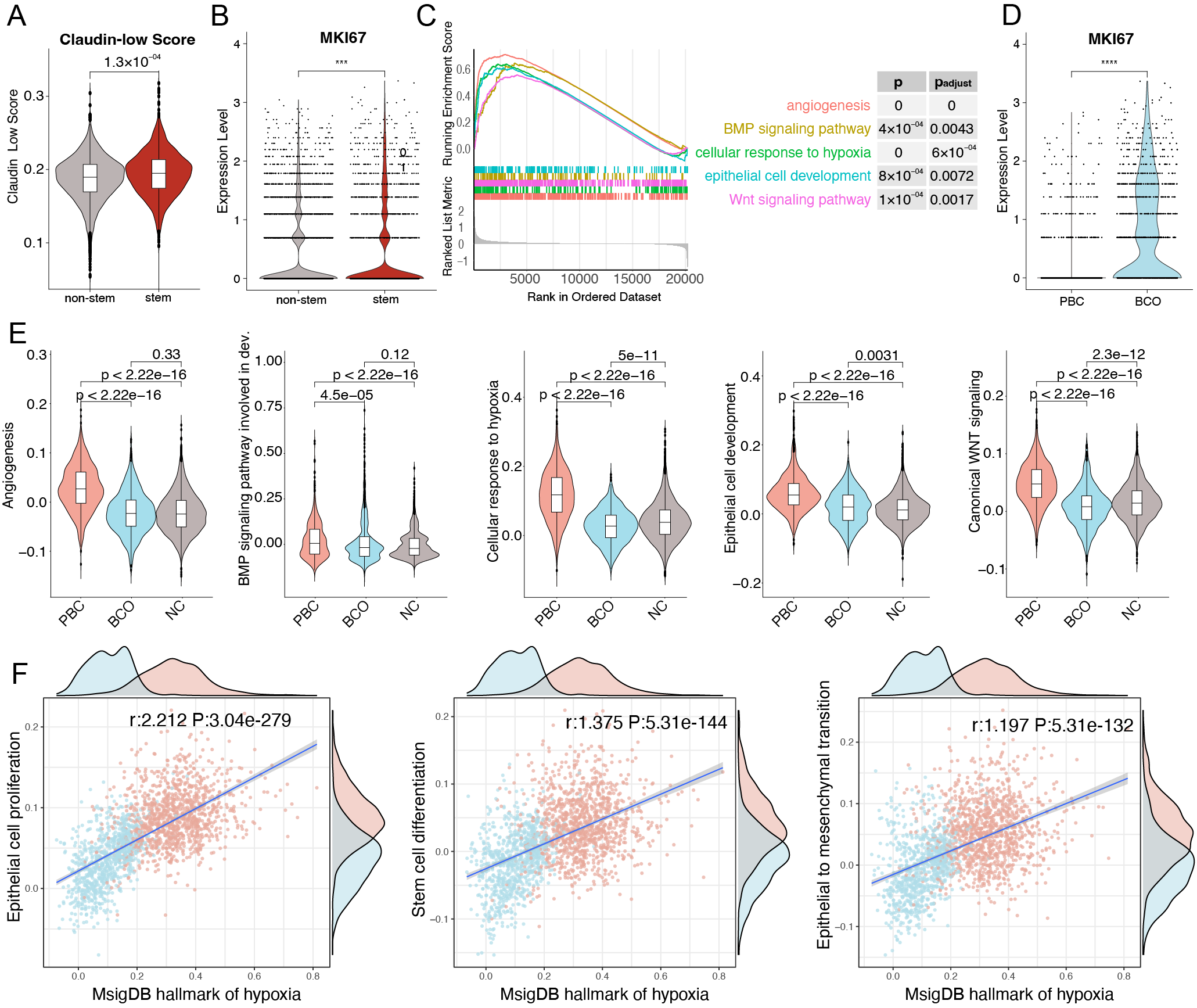


# Figure S6. Further function analysis on cancer stem cells in TNBC.

(**A**) Violin plot showing the comparison of MKI67 expressions between cells with or without stemness. (**B**) Violin plot showing the comparison of score of clawdin-low score between cells with or without stemness. (**C**) Violin plot showing the comparison of MKI67 expressions between stem cells from different sources. (**D**) GSEA enrichment plot for the differential expression genes between cells with or without stemness. (**E**) Violin plot showing the comparison of different function scores between cells of non-stem cells, stem cells from primary tumor, and stem cells from organoids. (**F**) Scatter plot and linear regression hypoxia hallmarker score and stem cell division score in TNBC cancer stem cells; Density plot demonstrating distribution of cells from BCO (blue) or BCA (red) are in upper and right. r stands for Pearson correlation ecoefficiency while P-value was calculated by linear regression t-test.

Abbreviations: PBC, primary breast cancer; BCO, breast cancer organoid; NC, non-hypoxia malignant cells; MsigDB, The Molecular Signatures Database.
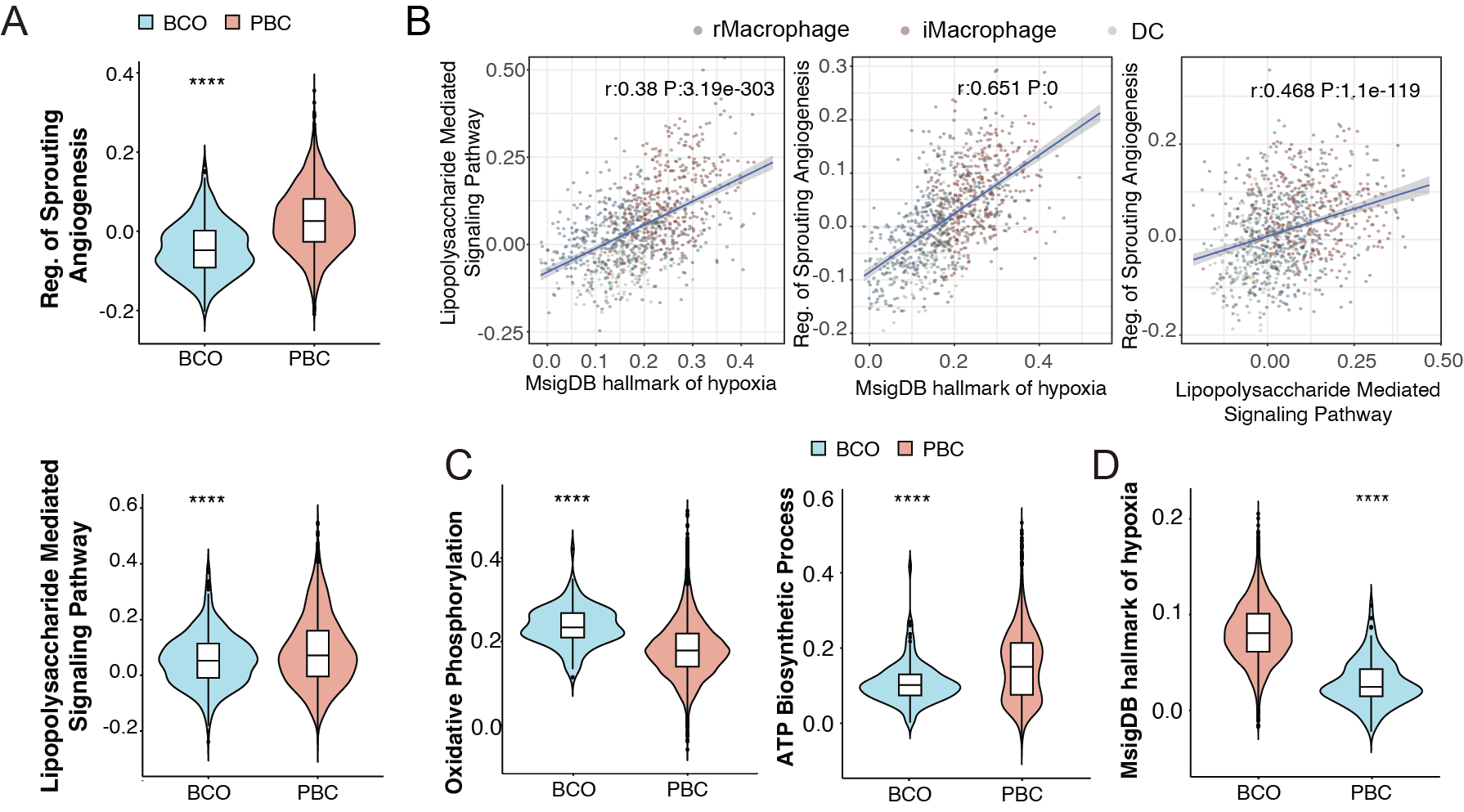


# Figure S8. Function analysis on hypoxia for tumor microenvironment in triple-negative breast cancer.

(**A**) Violinplot illustrating comparisons on scores of angiogenesis regulation and lipolyaccharide-mediate pathway between primary tumor samples (red) and organoid (blue). (**B**) Scatter plot and linear regression between function scores in myeloid cells and colors indicate myeloid cell types; r stands for Pearson correlation ecoefficiency while P-value was calculated by linear regression t-test. (**C**) Violin plot demonstrating energy-related functional scores in different cell subgroups. Asterisks stand for *P* values determined by Student’s t-tests between energy-related scores in T cells from PBC or BCO. (**D**) Violin plot showing hypoxia hallmark score in T cells from PBC or BCO; Asterisk stands for *P* values determined by Student’s t-tests between hypoxia score from PBC and BCO.


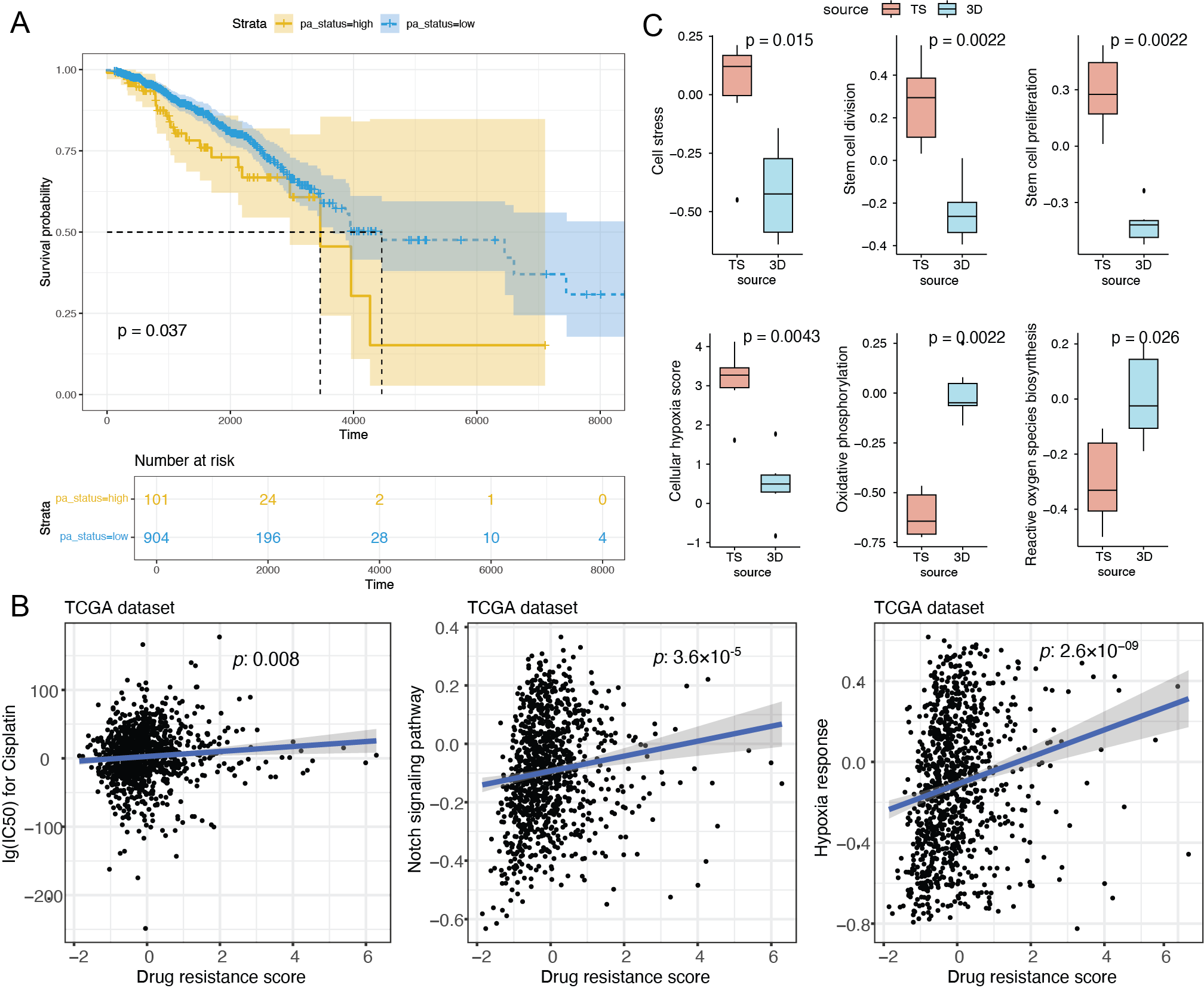


# Figure S8. Drug resistance validation with external database

(**A**) KM curve based on drug resistance score in TCGA. (**B**) Validation of the drug resistance score by correlation analysis between the score and cisplatin sensitivity, Notch signaling pathway, and hypoxia response. (**C**) Validation of expression patterns of key features between cancer tissue and 3D culture in outer database.

Abbreviations: TS: cancer tissue; 3D: 3D bioculture.


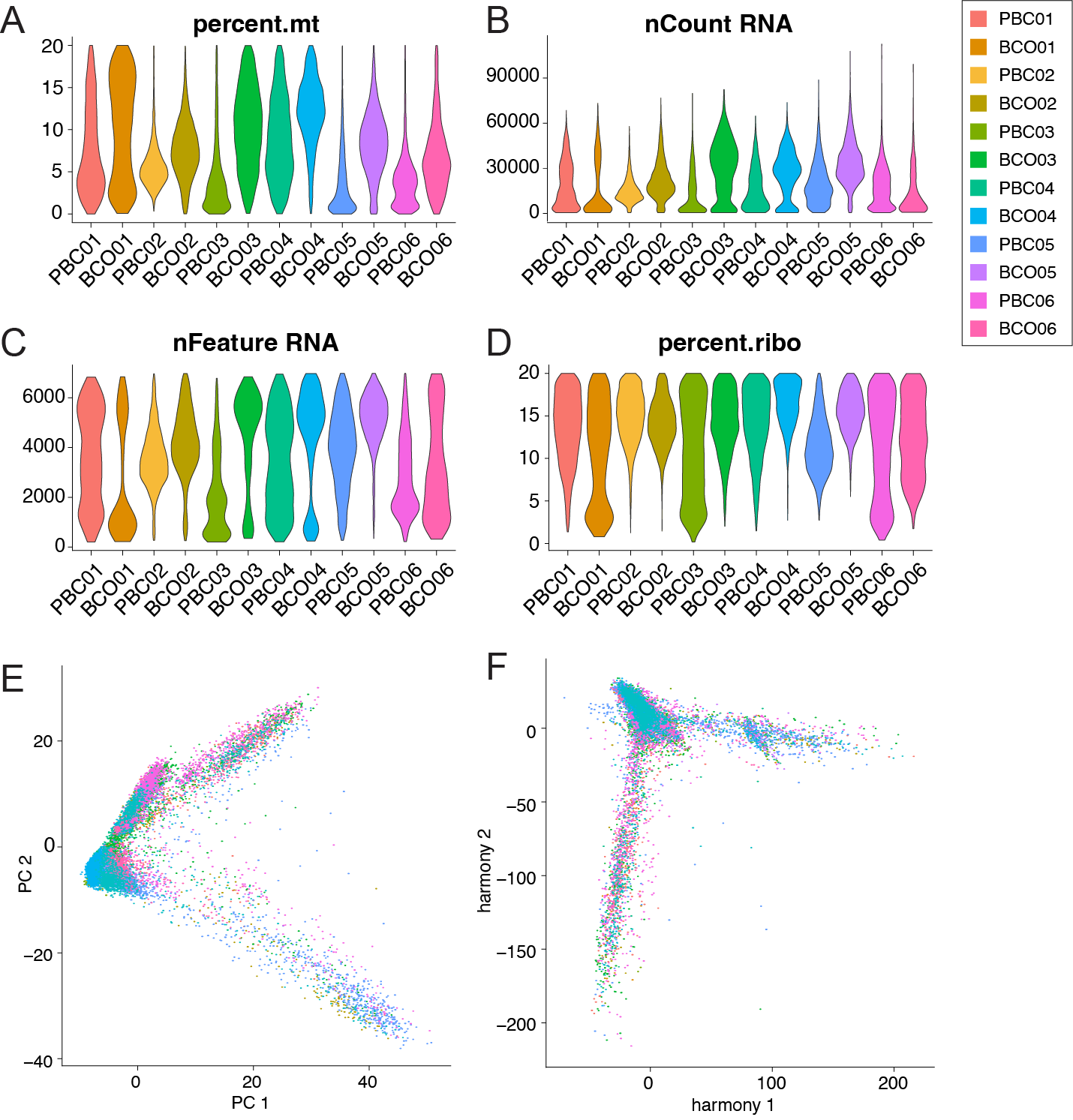


# Figure S9. Single-cell data quality control and batch elimination. (Cited in Supplementary Methods)

(**A**) Violin plot showing the mitochondrial gene proportion in each sample. PCA dimensionality reduction of cells and colors represent for samples. (**B**) Violin plot showing the number of counts in each sample. (**C**) Violin plot showing the number of features of RNA in each sample. (**D**) Violin plot showing the percentage of ribosome genes in each sample. (**E-F**) Dimensionality reduction of cells before (F) and after (G) harmony batch eliminations and colors represent for samples.

Abbreviations: PBC, primary breast cancer; BCO, breast cancer organoid; PCA, principal component analysis; percent.mt, percent of mitochondria gene expression; nCount_RNA, number of RNA counts; nFeature_RNA, number of genes; percent.ribo, percent of ribosome gene expression.


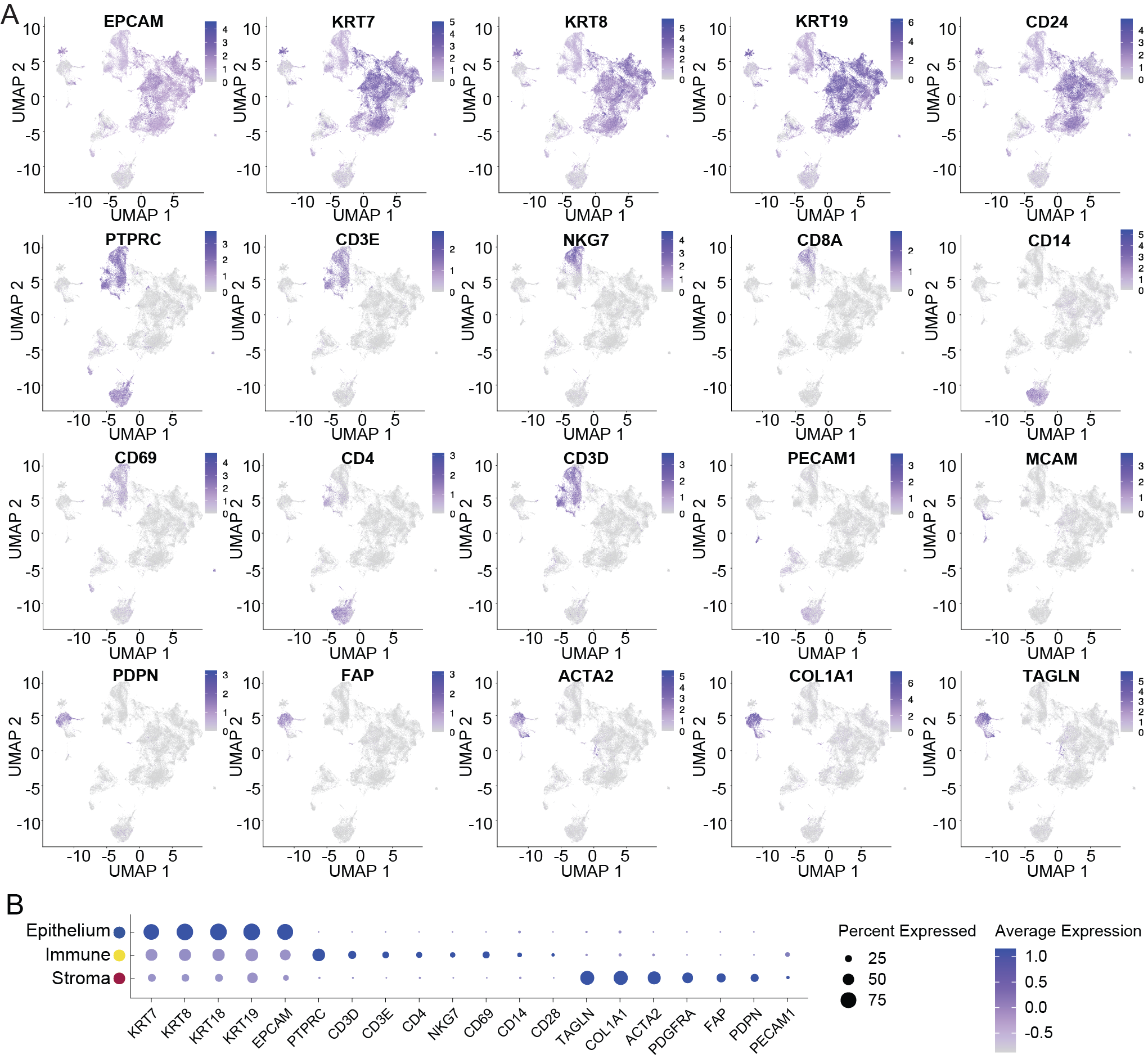


# Figure S10. Cell type annotation. (Cited in Supplementary Methods)

(**A**) UMAP dimensionality reduction of cells and colors represent gene expressions. (**B**) Expression patterns of different benign cell markers on each cell types.


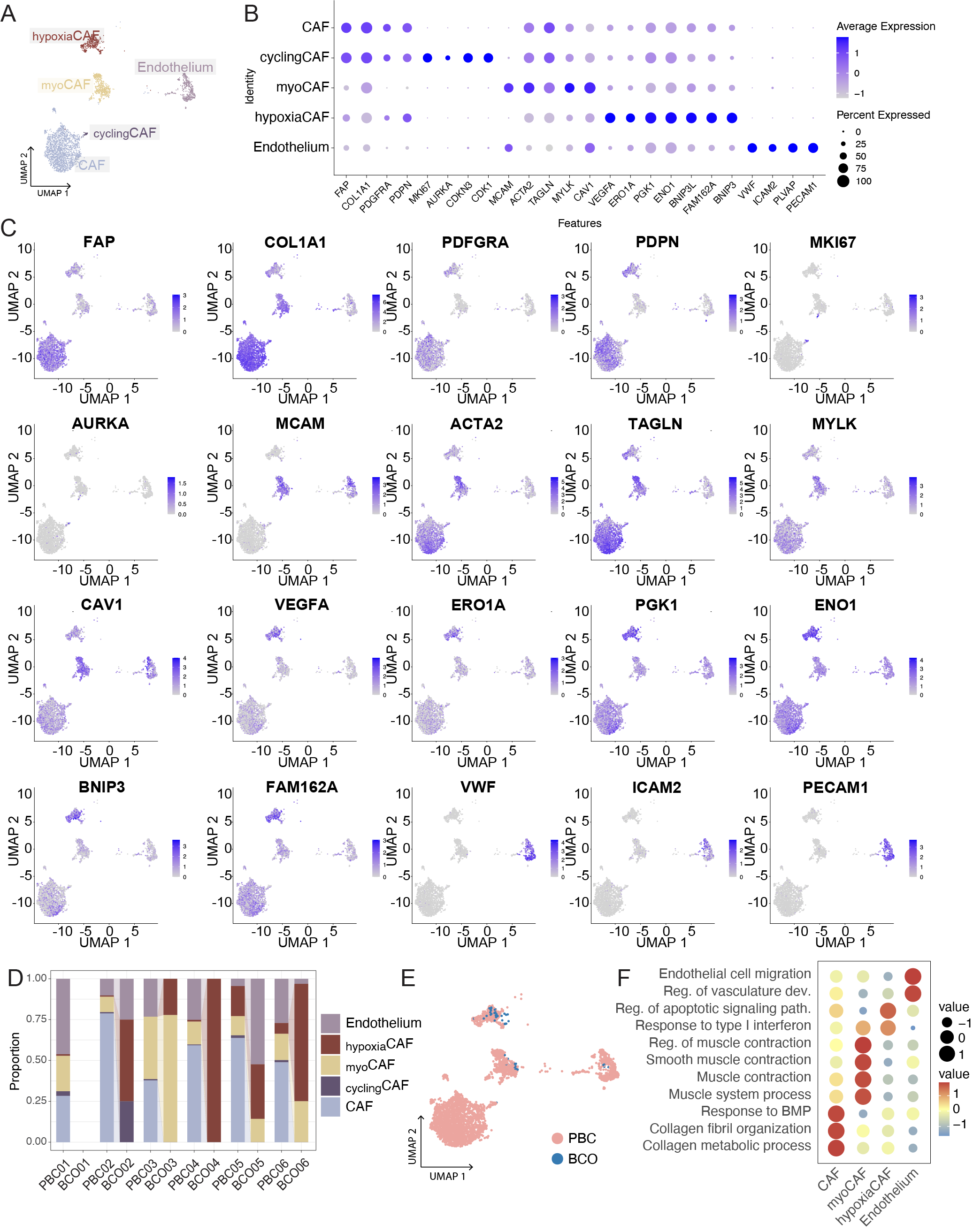


# Figure S11. Stromal cells in primary breast cancer and breast cancer organoids. (Cited in Supplementary Methods)

(**A**) UMAP dimensionality reduction of stromal cells and colors indicate stromal cell types. (**B-C**) Expression patterns of different stromal cell markers on each subgroup. (**D**) Stromal cell type ratios for primary tumor samples and organoids. Colors represent stromal cell types. (**E**) UMAP dimensionality reduction of stromal cells and colors represent sample type (primary tumor or organoids). (**F**) Scores and proportions of different stromal cell functions on each myeloid cell subgroups.

Abbreviations: PBC, primary breast cancer; BCO, breast cancer organoids; UMAP, uniform manifold approximation and projection; CAF, cancer-associated fibroblast.
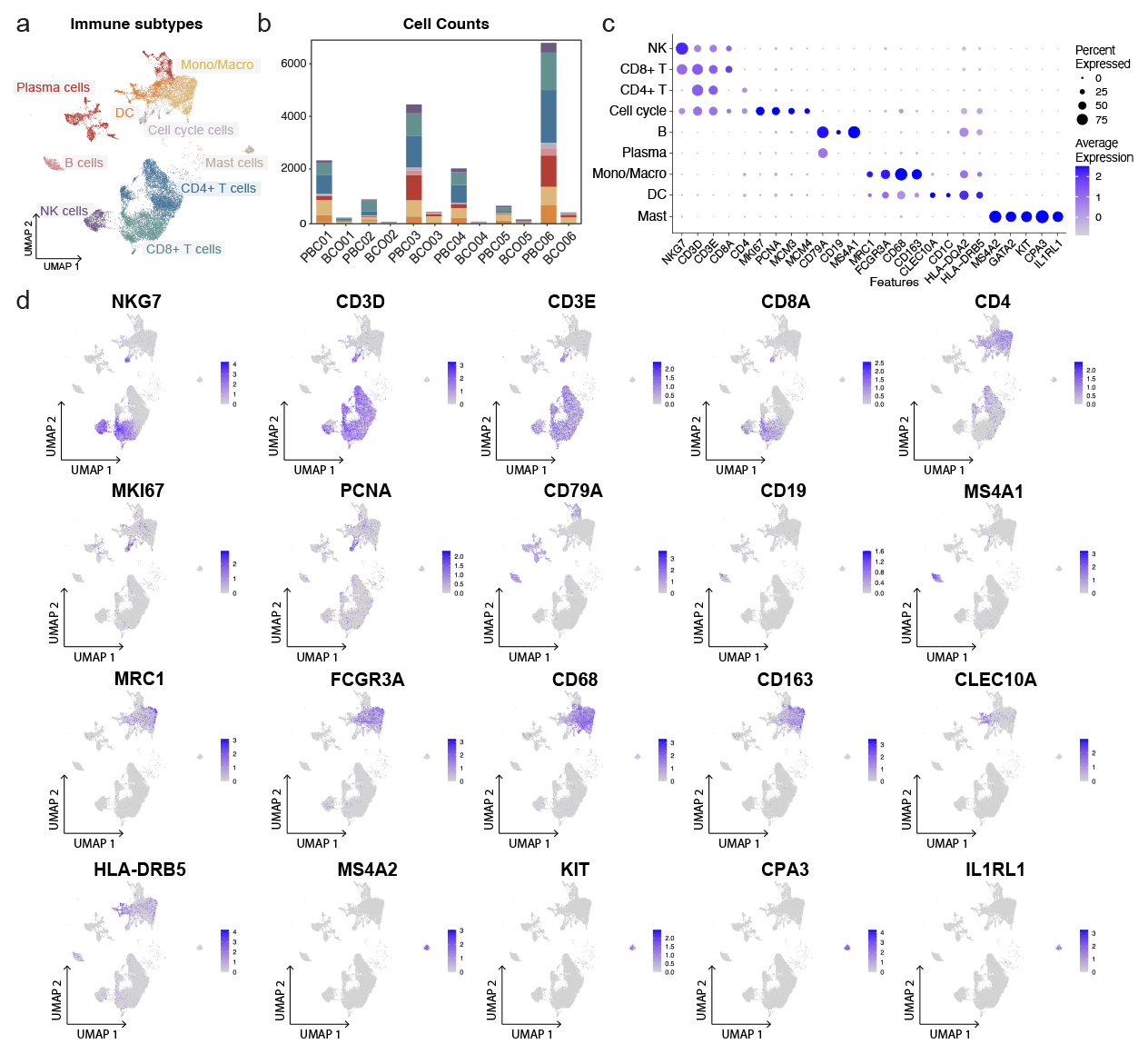


# Figure S12. Patient-derived organoids preserved immune cells of different subtypes.

(**A**) UMAP dimensionality reduction of immune cells and colors indicate immune cell types. (**B**) Immune cell type ratios for primary tumor samples and organoids. Colors represent immune cell types. (**C-D**) Expression patterns of different immune cell markers on each subgroup.

Abbreviations: PBC, primary breast cancer; BCO, breast cancer organoid; UMAP, uniform manifold approximation and projection; NK, natural killer cell; Mono, monocyte; Macro, macrophage; DC, dendritic cell.
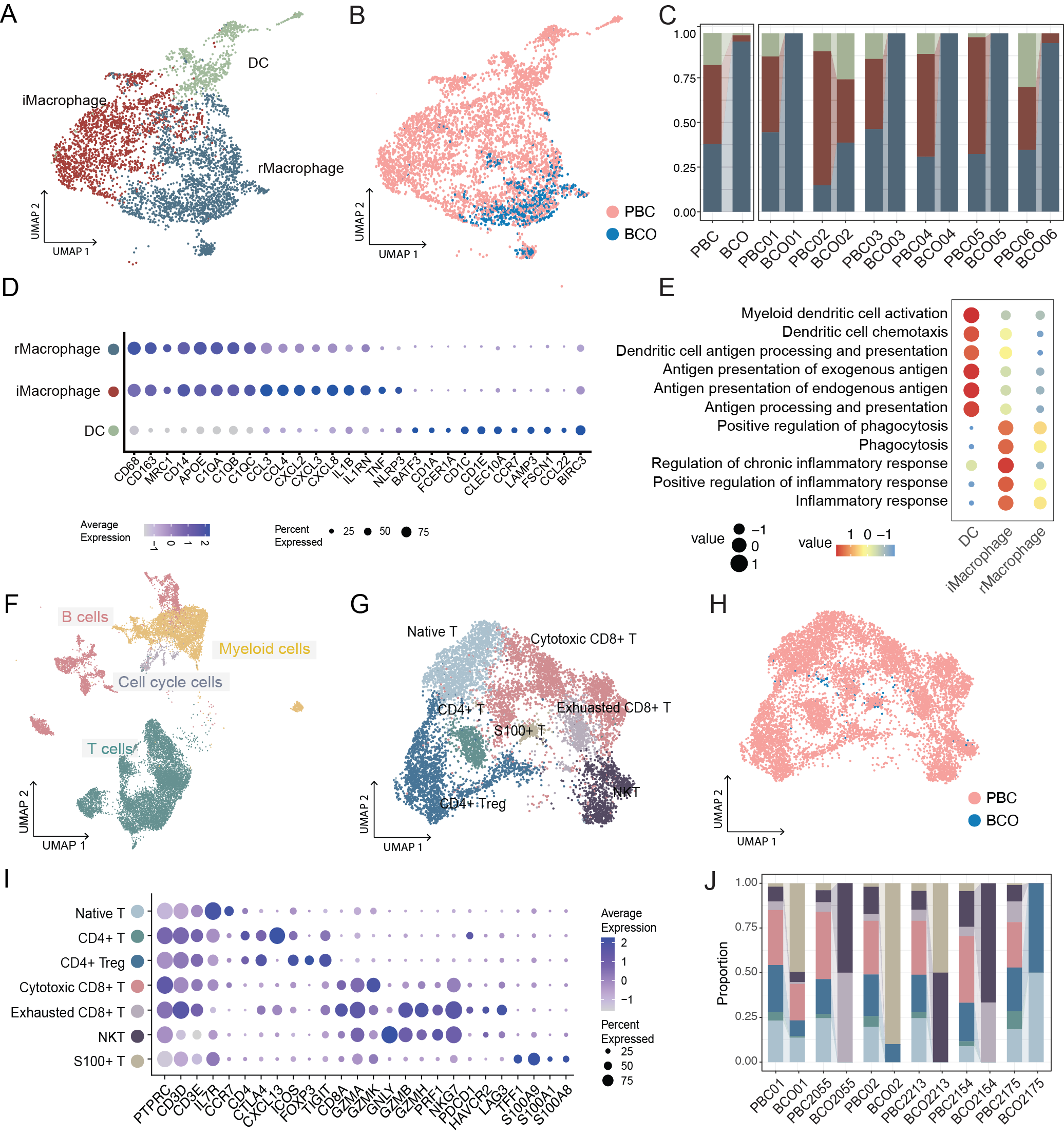


# Figure S13. Patient-derived organoids preserved immune cells with reduced quantity and functions.

(**A-B**) UMAP dimensionality reduction of myeloid cells and colors indicate myeloid cell types (A) or cell source (B). (**C**) Myeloid cell type ratios for primary tumor samples and organoids. Colors represent myeloid cell types. (**D**) Expression of different myeloid cell markers for different myeloid cell types. (**E**) Function bubble heatmap showing myeloid cell functions in different myeloid cell types. (**F**) UMAP dimensionality reduction of immune cells after standard preprocessing. (**G-H**) UMAP dimensionality reduction of T cells and colors indicate T cell subtypes (G), and sample source of PBC or BCO (H). (**I**) Expression of different T cell markers for different T cell subtypes. (**J**) T cell subtype ratios for primary tumor samples and organoids. Colors represent T cell subtypes.

Abbreviations: PBC, primary breast cancer; BCO, breast cancer organoid; DC, dendritic cell; iMacrophage, inflammatory macrophage; rMacrophage, resting macrophage; UMAP, uniform manifold approximation and projection; MsigDB, The Molecular Signatures Database.


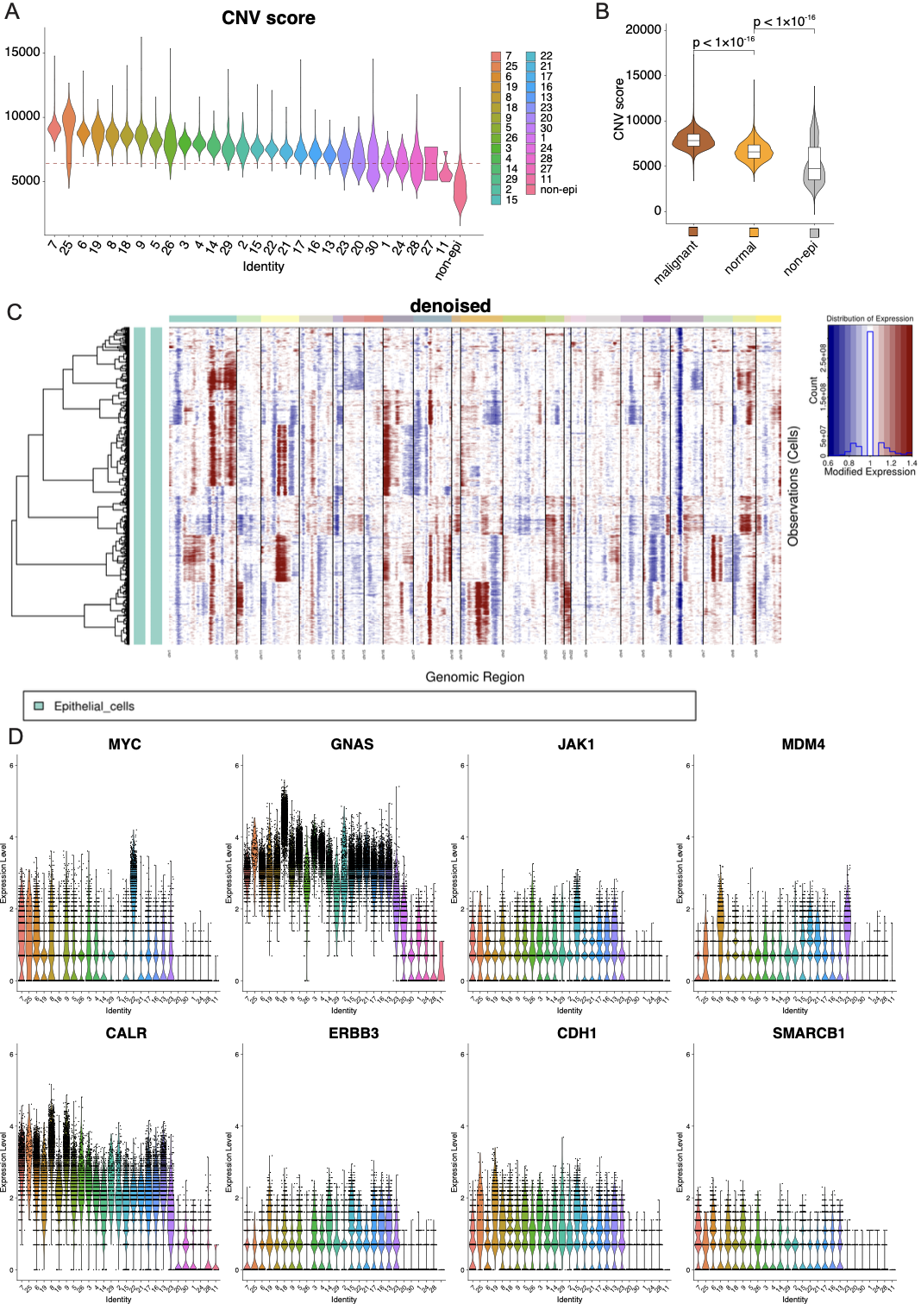


# Figure S14. Benign-malignant distinction of epithelium cells. (Cited in Supplementary Methods)

(**A**) Violin plot showing the distribution pattern of CNV scores in different clusters, sorted decreasingly by mean CNV scores. (**B**) Violin plot showing the comparison of CNV scores between malignant cells and benign cells. **(C**) Heatmap showing the overall CNV pattern of epithelium cells. (**D**) Key oncogenes expression level in different clusters.

Abbreviations: CNV, copy number variation; non-epi, non-epithelium cells.


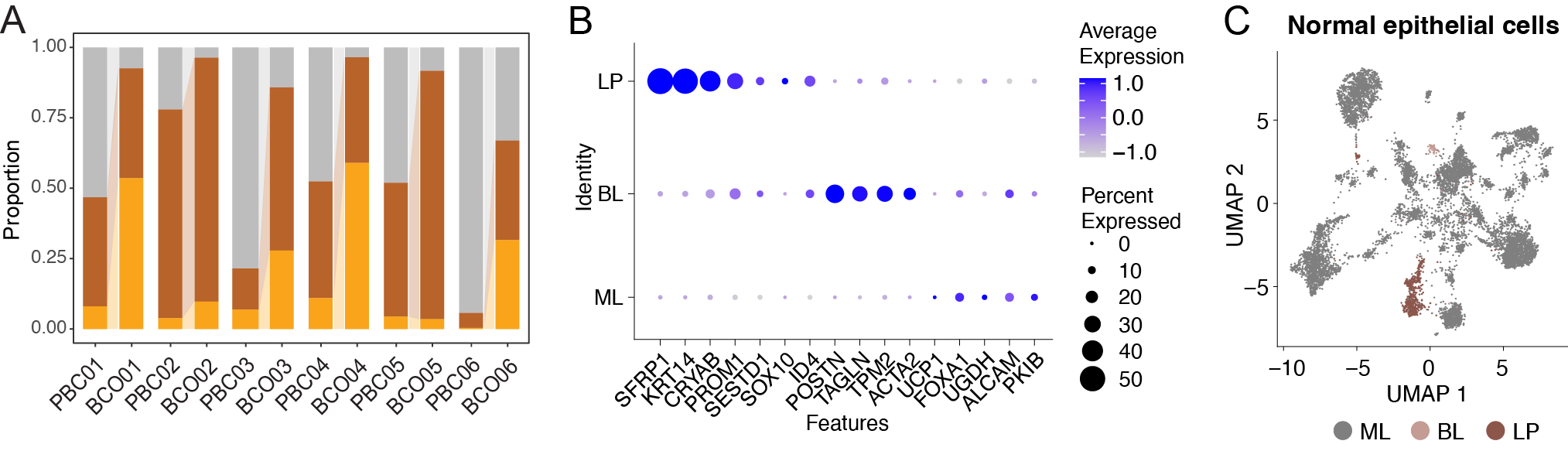


# Figure S15. Analysis on benign epithelium cells. (Cited in Supplementary Methods)

(**A**) Cell type ratios for primary tumor samples and organoids. Colors represent cell malignancy. (**B**) Expression patterns of different benign cell markers on each benign cell subgroups. (**C**) UMAP dimensionality reduction of cells and colors represent normal mammary epithelial cell type.

Abbreviations: PBC, primary breast cancer; BCO, breast cancer organoid; LP, luminal progenitor epithelium; BL, basal luminal epithelium; ML, mature luminal epithelium; UMAP, uniform manifold approximation and projection


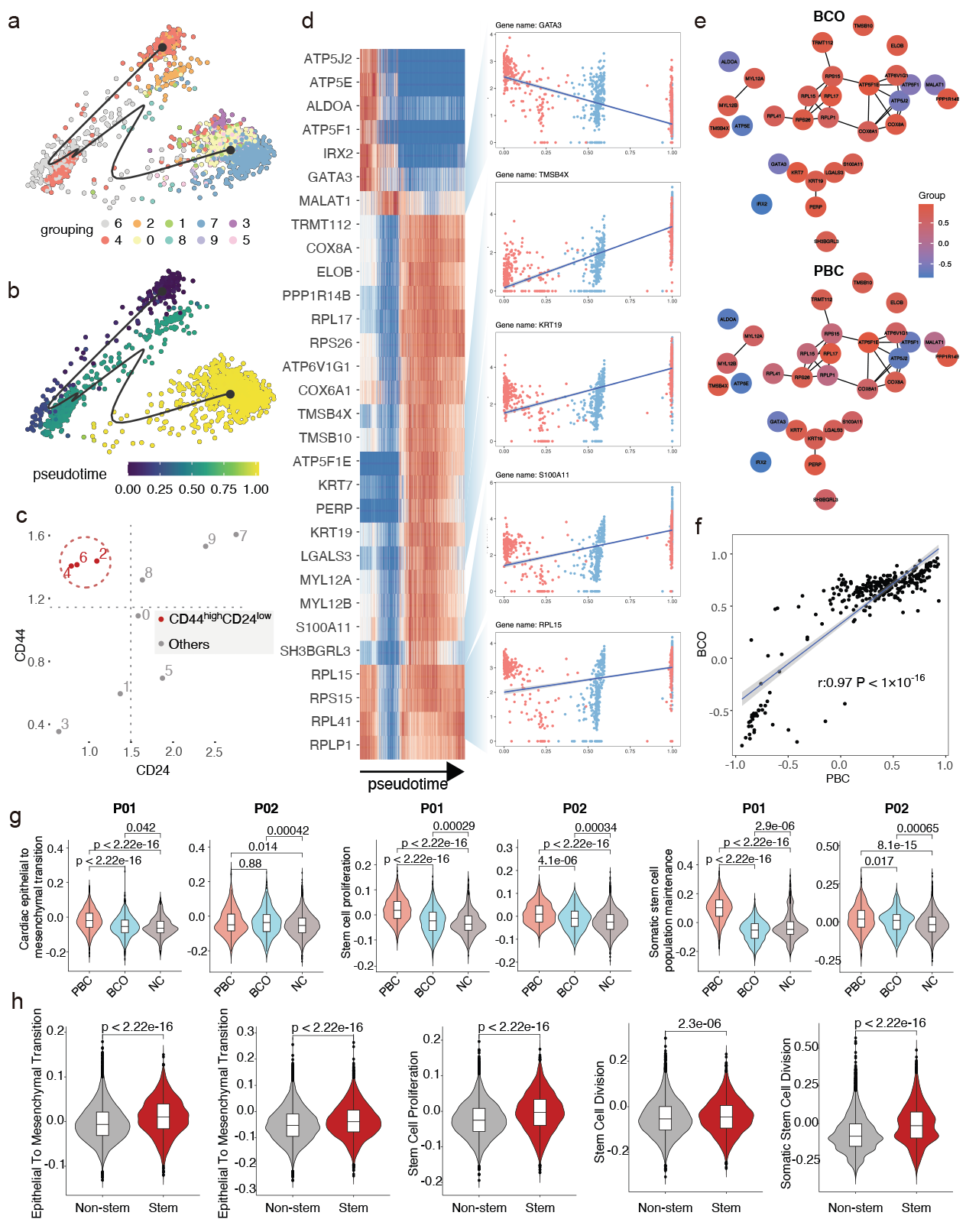


# Figure S16. Hormone receptor-positive breast cancer function analysis revealed preserved cell stemness and cell trajectory in tumor organoids.

(**A**) UMAP dimensionality reduction of ER+ malignant cells. Colors represent for cell subgroups. (**B**) UMAP dimensionality reduction of ER+ malignant cells. Colors represent for pesudotime. (**C**) Dot plot showing the expression of CD44 and CD24 in each cell cluster. (**D**) Expression patterns for selected trajectory-important genes in ER+ malignant cells. (**E**) Gene regulatory network for selected trajectory-important genes in primary tumor and matched organoids. Colors represent for Pearson correlation coefficients (PCCs) between gene expressions and pesudotime scores. (**F**) Dot plot showing the positive Pearson correlation of PCCs in panel E between primary tumor and matched organoids. (**G**) Violin plot showing the comparison of different function scores between cells of non-stem cells, stem cells from primary tumor, and stem cells from organoids. (**H**) Violin plot showing the comparison of score of stemness-related pathways between cells with or without stemness.

Abbreviations: PBC, primary breast cancer; BCO, breast cancer organoid; UMAP, uniform manifold approximation and projection; PCC, Pearson correlation coefficient; ER+, estrogen receptor positive subtype.


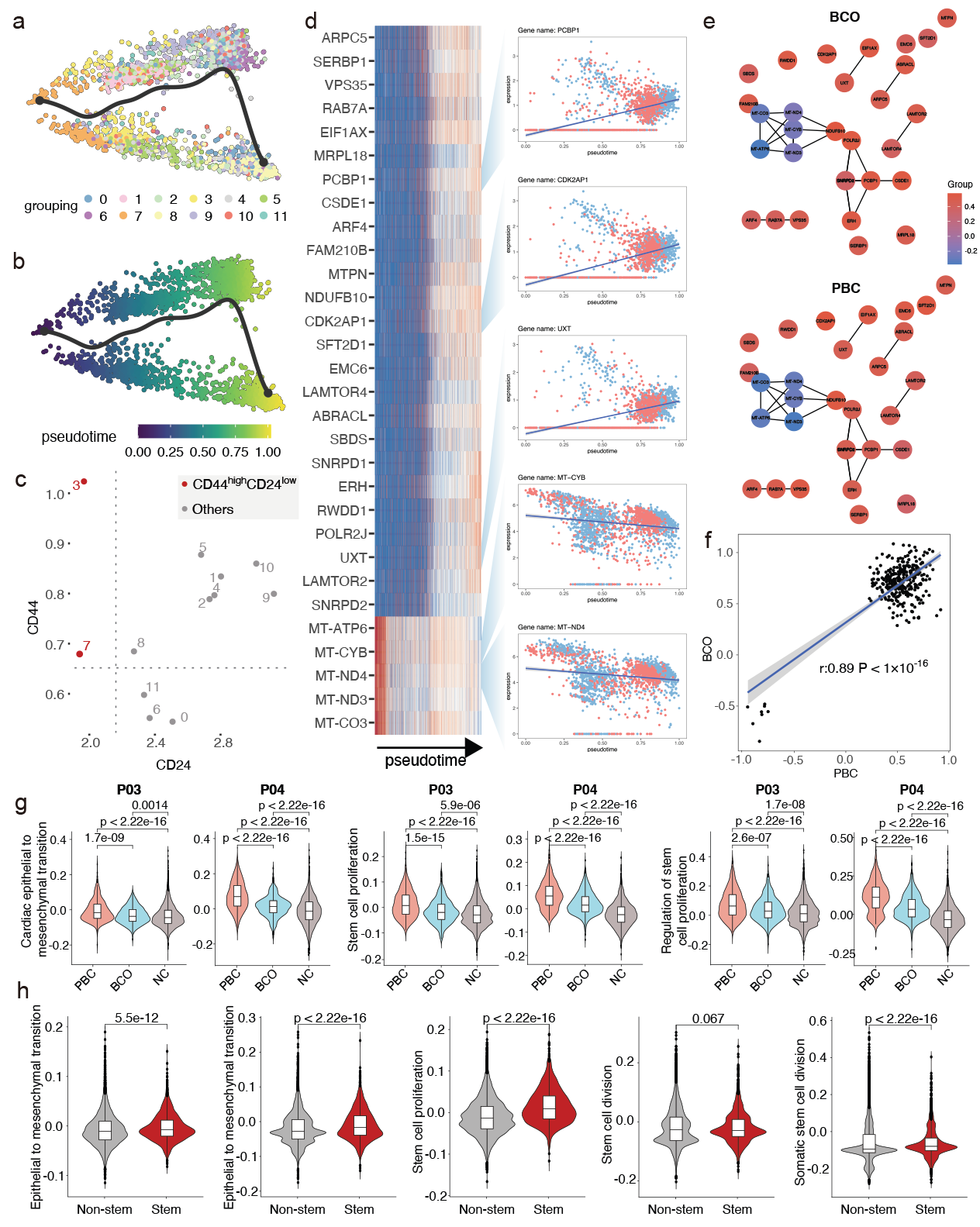


# Figure S17. HER2-positive breast cancer function analysis revealed preserved cell stemness and cell trajectory in tumor organoids.

(**A**) UMAP dimensionality reduction of HER2+ malignant cells. Colors represent for cell subgroups. (**B**) UMAP dimensionality reduction of HER2+ malignant cells. Colors represent for pesudotime. (**C**) Dot plot showing the expression of CD44 and CD24 in each cell cluster. (**D**) Expression patterns for selected trajectory-important genes in HER2+ malignant cells. (**E**) Gene regulatory network for selected trajectory-important genes in primary tumor and matched organoids. Colors represent for Pearson correlation coefficients (PCCs) between gene expressions and pesudotime scores. (**F**) Dot plot showing the positive Pearson correlation of PCCs in panel E between primary tumor and matched organoids. (**G**) Violin plot showing the comparison of different function scores between cells of non-stem cells, stem cells from primary tumor, and stem cells from organoids. (**H**) Violin plot showing the comparison of score of stemness-related pathways between cells with or without stemness.

Abbreviations: PBC, primary breast cancer; BCO, breast cancer organoid; UMAP, uniform manifold approximation and projection; PCC, Pearson correlation coefficient; HER+, HER2 positive subtype.

# Figure S18. Global CNV patterns in malignant epithelium cells

(**A**) UMAP dimensionality reduction of malignant cells from patient 03 with an inferred trajectory. Colors represent pesudotime. (**B**) Heatmap showing the dominant CNV in 44 chromosome regions for each cell subclusters in patient 03 malignant cells. (**C**) Heatmap for inferred CNV on malignant cells for patient 03 and colors represent for amplifications or deletions. (**D-E**) Heatmap showing the dominant CNV in 44 chromosome regions for each cell subclusters in the other five patients’ malignant cells.

Abbreviations: CNV, copy number variation; chr, chromosome; q, long arm; p, short arm; UMAP, uniform manifold approximation and projection.
